# Nanotherapeutics for Alleviating Anesthesia‐Associated Complications

**DOI:** 10.1002/advs.202308241

**Published:** 2024-02-11

**Authors:** Bin Lu, Ling Wei, Gaoxiang Shi, Jiangfeng Du

**Affiliations:** ^1^ Department of Anesthesiology Third Hospital of Shanxi Medical University Shanxi Bethune Hospital Shanxi Academy of Medical Sciences Tongji Shanxi Hospital Taiyuan 030032 China; ^2^ Key Laboratory of Cellular Physiology at Shanxi Medical University Ministry of Education Taiyuan Shanxi Province 030001 China; ^3^ Shanxi Bethune Hospital Center Surgery Department Shanxi Academy of Medical Sciences Tongji Shanxi Hospital Third Hospital of Shanxi Medical University Taiyuan 030032 China; ^4^ Department of Medical Imaging Shanxi Key Laboratory of Intelligent Imaging and Nanomedicine First Hospital of Shanxi Medical University Taiyuan Shanxi Province 030001 China

**Keywords:** anesthesia, complication, detoxification, nanomedicine

## Abstract

Current management of anesthesia‐associated complications falls short in terms of both efficacy and safety. Nanomaterials with versatile properties and unique nano‐bio interactions hold substantial promise as therapeutics for addressing these complications. This review conducts a thorough examination of the existing nanotherapeutics and highlights the strategies for developing prospective nanomedicines to mitigate anesthetics‐related toxicity. Initially, general, regional, and local anesthesia along with the commonly used anesthetics and related prevalent side effects are introduced. Furthermore, employing nanotechnology to prevent and alleviate the complications of anesthetics is systematically demonstrated from three aspects, that is, developing 1) safe nano‐formulization for anesthetics; 2) nano‐antidotes to sequester overdosed anesthetics and alter their pharmacokinetics; 3) nanomedicines with pharmacodynamic activities to treat anesthetics toxicity. Finally, the prospects and challenges facing the clinical translation of nanotherapeutics for anesthesia‐related complications are discussed. This work provides a comprehensive roadmap for developing effective nanotherapeutics to prevent and mitigate anesthesia‐associated toxicity, which can potentially revolutionize the management of anesthesia complications.

## Graphical abstract

Nanotechnology exhibits excellent performance in anesthesia‐associated toxicity via safety nano‐formulization design, alter their pharmacokinetics and higher pharmacodynamic activities of anesthetics toxicity.

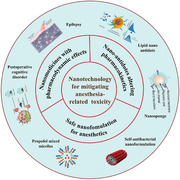

## 1 Introduction

Medieval surgical procedures conducted without anesthesia were characterized by extreme brutality. It was not until 1846 when dentist William T.G. Morton successfully demonstrated the first public general anesthesia with inhaled ether that brought humankind to the dawn of painless surgery.^[^ ^1^ ^]^ Over a span of 177 years, anesthesiology has transitioned from a medical discipline into a specialized field, with a primary mission of ensuring patient comfort and safety for related clinical practices.^[^ ^2^ ^]^ A prominent catalyst for the development of anesthesiology was the inadequacy of early anesthetics. For example, diethyl ether exhibited substantial limitations, including high flammability, slow onset of action, a high incidence of severe nausea and vomiting, and an unpleasant odor.^[^ ^1^ ^]^ Although contemporaneous chloroform displayed improved comfort and potency characteristics, its use led to the first recorded death from anesthesia in 1848, followed by up to fifty deaths by 1858.^[^ ^2^, ^3^ ^]^ Consequently, chloroform serves as a poignant reminder of the imperative balance between anesthetics potency and patient safety when utilizing anesthetics.

To date, extensive research has yielded a myriad of anesthetics with high efficacy and low mortality and morbidity rates, including sevoflurane, propofol, ketamine, etomidate, opioids, and lidocaine.^[^ ^2^ ^]^ Nevertheless, these anesthetics still pose risks to susceptible patient populations or under erroneous implementation. For instance, the concomitant use of volatile anesthetics and succinylcholine in emergency situations can potentially trigger lethal malignant hyperthermia (MH) in vulnerable individuals.^[^ ^4^, ^5^, ^6^ ^]^ Opioids, renowned for their indispensable analgesic effects, exhibit tolerance and addiction potential upon repeated administration and can induce life‐threatening respiratory depression in cases of overdose.^[^ ^7^ ^]^ Misapplication of local anesthetics can result in severe systemic toxicity, affecting the central nervous system (CNS) and cardiovascular system.^[^ ^8^ ^]^ Therefore, preventing and managing anesthetics‐related toxicity remain formidable challenges during the perioperative period.

Nanotechnology, characterized by its versatile properties, presents unprecedented opportunities for addressing anesthetics‐related complications and enhancing the safety of anesthesia practice. First, nano‐formulation of anesthetics/therapeutics can potentially overcome challenges associated with drug stability, solubility, and bioavailability, while simultaneously eliminating the use of hazardous pharmaceutical excipients.^[^ ^9^, ^10^, ^11^ ^]^ Moreover, adaptable nanostructures can be engineered to efficiently sequester overdosed anesthetics, subsequently altering their biodistribution, metabolism, and excretion profiles to mitigate toxicity.^[^ ^12^, ^13^ ^]^ Furthermore, owing to unique nano‐bio interactions, nanomedicines can achieve targeted accumulation and on‐demand release in vital organs such as the brain, spinal cord, liver, and kidney, facilitating the treatment of anesthetics‐related neurotoxicity, hepatotoxicity, and nephrotoxicity.^[^ ^14^, ^15^, ^16^ ^]^ Notably, nanomaterials exhibiting suitable mechanical, electronic, biochemical, topological and engineering features hold promise as neural tissue scaffolds, offering multifaceted clues to guide the regeneration of injured spinal cord resulting from regional anesthesia.^[^ ^17^, ^18^, ^19^ ^]^ From a prophylactic perspective, nano‐biosensors lay foundation for accurate, sensitive, and real‐time detection of anesthetics or related biomolecules, aiding safe anesthesia practice and enabling investigations into the mechanisms of anesthetics‐related toxicity.^[^ ^20^, ^21^ ^]^

**Scheme 1:**
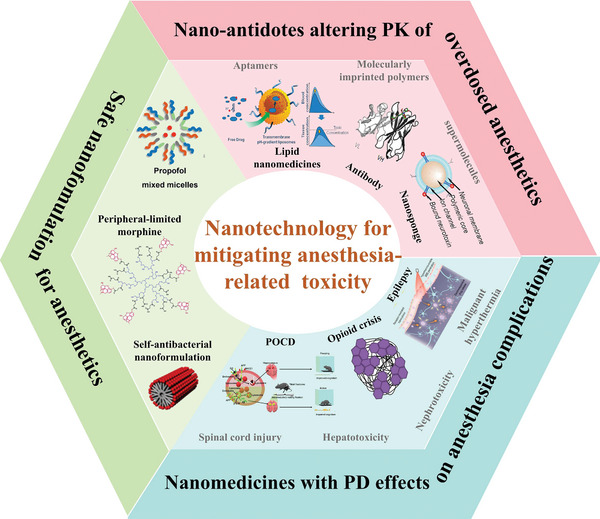
Strategies of employing nanotechnology to mitigate complications associated with anesthesia.

Despite the severity and frequency of anesthetics‐related toxicity, current research efforts and progression in managing these complications remain limited. The advent of nanotechnology is poised to introduce significant advancements in this critical field. Therefore, this manuscript provides a comprehensively review of the existing nanotherapeutics and underscores the strategies for developing prospective nanomedicines to ameliorate anesthetics‐related toxicity. Initially, we introduce the frequently used modern anesthetic agents for general, regional, and local anesthesia, along with their prevalent hazardous effects. Subsequently, we systematically elaborate on how nanotechnology can be employed to prevent and alleviate anesthetic complications from three perspectives (**Scheme** **1**): 1) Nano‐formulization of anesthetics to eliminate the usage of irritating excipients; 2) Nano‐antidotes to sequester overdosed anesthetics and alter their pharmacokinetics (PK); 3) Nanomedicines with pharmacodynamic (PD) effects to treat the symptoms of anesthetics toxicity. Finally, the prospects and obstacles regarding the clinical translation of nanotherapeutics for managing anesthetics toxicity are discussed.

## 2 Modern Anesthesia and Related Toxicity

There are primarily three types of anesthesia practice utilized both in and out of the operating room: general anesthesia, regional anesthesia (RA), and local anesthesia (LA). These anesthesia modalities can be employed individually or in combination based on various factors such as the type of surgery, patient conditions, and potential drug interactions.^[^ ^22^, ^23^ ^]^ Even though anesthesia‐related adverse events have declined over decades, there is still an estimated 18%–20% incidence rate of temporary or permanent damages associated with anesthesia.^[^ ^24^ ^]^ The incidences of complications after anesthesia often present relatively large variability, which depends on several covariates, including the types of operation, anesthesia techniques and drugs, time after surgery, and patient characteristics (e.g., age, sex, race, comorbidities).^[^ ^25^, ^26^ ^]^ This section aims to provide an overview of modern anesthesia and associated complications (**Table** **1**).

**Table 1:** The primary anesthesia practices, commonly used anesthetic agents and related complications.

Type of anesthesia	Category of anesthetics	Commonly used anesthetic agents or techniques and their complications
General anesthesia	Common complications: laryngospasm, apnea, postoperative nausea and vomiting (PONV), hypotension, and short‐term neurocognitive disorders	
	Volatile anesthetics	Nitrous oxide, halogenated alkanes: malignant hyperthermia, hepatotoxicity, nephrotoxicity
	Intravenous anesthetics a) Sedative‐hypnotic agents	Propofol: bradycardia, hypotension, propofol‐related infusion syndrome (PRIS) Etomidate: high PONV incidence, hiccoughs, myoclonus, adrenal suppression, injection pain, hyperglycemia, acute tubular necrosis, metabolic acidosis, Epilepsy Midazolam, diazepam: neuropsychiatric disturbance Ketamine: PONV, psychomimetic events, hallucinations, delirium, and hypertension, epilepsy
	b) Analgesics	Opioids: CNS depression, nausea, vomiting, constipation, pruritus, respiratory suppression, urinary retention, opioid tolerance and hyperalgesia, dependence, withdrawal symptoms, addiction, death Ketamine: see above
	c) Muscular relaxants	Suxamethonium, pancuronium, etc.: hypoxemia, airway obstruction, allergic and pseudo‐allergic reactions
Regional anesthesia	Local anesthetics	Neuraxial anesthesia: post‐dural puncture headache, hemorrhage, cord compression, paraplegia, neurological infections, nerve injuries; Peripheral nerve block: nerve injuries, infection, bleeding, pain Toxicity associated with local anesthetics (lidocaine, bupivacaine, ropivacaine): systemic toxicity (LAST, mainly central neurotoxicity and cardiorespiratory toxicity), allergic reactions, methemoglobinemia
Local anesthesia	Local anesthetics	Nerve injuries, infection, bleeding, pain, LAST, allergic reactions

### 2.1 General Anesthetics and Related Toxicity

General anesthesia with endpoints of hypnosis, amnesia, analgesia and immobility is essential for major surgical procedures to ensure the smooth progress of operations and the comfort and safety of patients.^[^ ^2^, ^27^ ^]^ Modern general anesthesia adopts the concept of balanced anesthesia, utilizing various types of anesthetic agents synergistically to enhance effectiveness and reduce morbidity.^[^ ^22^, ^28^ ^]^ Various inhalational anesthetics and intravenous anesthetics are used for inducing and maintaining general anesthesia. Hazardous effects of anesthetics are one of the most accountable factors for anesthesia complications. Possible adverse events after general anesthesia encompass common complications (e.g., postoperative nausea and vomiting (PONV), laryngospasm, hypotension, and temporary neurocognitive disorders), and less frequent but potentially disastrous complications, which include respiratory failure, myocardial infarction, renal failure, and in extreme cases, death.^[^ ^24^, ^29^, ^30^ ^]^ In detail, a meta‐analysis revealed that the global prevalence of PONV was 27.5% with an range between 6.7% and 73.4%.^[^ ^26^ ^]^ Laryngospasm was found to frequently occur in pediatric patients with an incidence rate of 18.4%.^[^ ^25^ ^]^ The prevalence of intraoperative hypotension ranges from 5% to 99% with 41% of patients experiencing any episode of systolic blood pressure < 80 mmHg.^[^ ^31^ ^]^ Depending on the cognitive assessment time, postoperative cognitive dysfunction has an incidence range of 25% to 40% one week after surgery, reducing to ≈10% three months post‐surgery.^[^ ^32^, ^33^, ^34^ ^]^ Among 69 754 patients receiving general anesthesia for lower extremity amputation, ≈6.7%, 1.5%, 2.0% of them experienced respiratory failure, myocardial infarction, and renal failure, respectively.^[^ ^30^ ^]^

#### 2.1.1 Inhalational Anesthetics and Related Toxicity

Inhalational anesthetics currently in clinical use primarily include nitrous oxide (N_2_O) and halogenated alkanes. N_2_O, with low anesthetic potency, is often combined with halogenated anesthetics. Halogenated alkanes, derived from chemical modifications of ether to reduce inflammability, constitute the largest group of volatile anesthetics, which includes halothane, enflurane, isoflurane, desflurane and sevoflurane.^[^ ^2^ ^]^

Different anesthetic drugs can lead to unique adverse events. For instance, N_2_O has the potential to induce hematologic, neurologic, and myocardial toxicity due to its inhibition of methionine synthase, a critical enzyme involved in nucleic acid synthesis and nerve myelination.^[^ ^35^, ^36^ ^]^ Patients who are seriously ill, elderly, alcoholic or deficient in cobalamin and/or folate are particularly susceptible to N_2_O‐related toxicity, especially with long‐term and high‐dose exposure to N_2_O.^[^ ^35^ ^]^ As the most commonly administered volatile anesthetics, halogenated alkanes can induce varying degrees of hepatotoxicity, which correlates with their respective levels of hepatic metabolism.^[^ ^37^ ^]^ The hepatic metabolism of halothane, enflurane, sevoflurane, isoflurane and desflurane was estimated to be 15%–20%, 1%–2%, 3%–4%, 0.2%, and 0.02%, respectively.^[^ ^37^, ^38^, ^39^ ^]^ The hepatic metabolite for the majority of halogenated anesthetics is trifluoroacetic acid (TFA) except for sevoflurane, which is metabolized into hexafluoroisopropanol (HFIP) and inorganic fluoride.^[^ ^38^, ^39^ ^]^ Unlike HFIP, TFA has a higher binding affinity for hepatic proteins and lipids, generating more immunogenic adducts and causing more severe liver damage.^[^ ^37^, ^39^ ^]^ While halothane is abandoned in developed countries, it remains a threat in developing countries with an incidence rate of 24.4%. Although the liver injury incidence of modern halogenated anesthetics is estimated to be ≈ 4%,^[^ ^40^ ^]^ long‐term occupational exposure to anesthetic gas waste can lead to alterations in hematological and hepatic functions.^[^ ^41^ ^]^ In addition, inorganic fluoride resulting from sevoflurane's metabolism and by‐products upon exposure to alkaline absorbents have been reported to be nephrotoxic.^[^ ^42^ ^]^

Almost all volatile anesthetics except for xenon have been recognized as risk factors for malignant hyperthermia (MH).^[^ ^43^, ^44^ ^]^ Despite the extremely low incidence of MH (i.e., 0.0006%–0.01%), it is a potentially fatal pharmacogenetic disorder of skeletal muscle tissue, particularly affecting patients with mutations in the ryanodine receptor or dihydropyridine receptor.^[^ ^44^ ^]^ MH is characterized by the rapid release of calcium ions from the sarcoplasmic reticulum into the cytoplasm of skeletal cells, resulting in cell hypermetabolism and its clinical manifestations such as tachycardia, muscle rigidity, hyperthermia, rhabdomyolysis, and acidosis.^[^ ^6^, ^45^ ^]^ With the introduction of dantrolene and improvements in early prediction, diagnosis and adjuvant treatments, the mortality rate associated with MH declined from 70% to 80% in the 1960s to <10% today.^[^ ^6^, ^46^ ^]^

#### 2.1.2 Intravenous Anesthetics and Related Toxicity

Intravenous anesthetic agents can be broadly categorized based on their PD actions as sedative‐hypnotic agents, analgesics, and muscular relaxants.^[^ ^2^, ^28^, ^47^ ^]^ Sedative‐hypnotic agents inhibit CNS functions to induce sedation, sleep, and unconsciousness with increasing doses. Commonly used sedatives for anesthesia include propofol, etomidate, benzodiazepines (BDZs), and ketamine.^[^ ^2^ ^]^ Among them, propofol is most frequently administered for inducing and maintaining anesthesia intravenously. Propofol has been reported to decrease PONV incidence but may increase the risk of short‐term cardiovascular complications such as bradycardia and hypotension.^[^ ^48^, ^49^ ^]^ Propofol‐related infusion syndrome (PRIS), a rare but potentially fatal condition caused by prolonged propofol infusion, results in excessive lipolysis and inhibited adenosine triphosphate (ATP) synthesis, leading to cardiovascular dysfunction and multiple organ failure.^[^ ^50^ ^]^ The incidence of PRIS in critically ill patients was estimated to be 2.9% with an associated mortality rate of 36.8%.^[^ ^51^ ^]^ Etomidate is a popular choice for anesthesia induction in patients with cardiac dysfunction due to its superior cardiovascular stability. However, etomidate administration is related to a high PONV incidence (30%–40%), hiccoughs, myoclonus and adverse events related to etomidate's solvent, such as injection pain, hyperglycemia, acute tubular necrosis and metabolic acidosis.^[^ ^2^, ^52^ ^]^ In severe cases, etomidate can lead to detrimental adrenal suppression and high mortality in patients with sepsis and critical illness.^[^ ^53^, ^54^ ^]^ Representatives of BDZs for anesthesia include midazolam, diazepam and remimazolam. BDZs are favored for preoperative and diagnostic/therapeutic sedation due to their relatively mild anesthetic profiles and limited adverse effects.^[^ ^55^, ^56^ ^]^ Among them, remimazolam, characterized by rapid onset and elimination, is advantageous over midazolam, which is associated with prolonged neuropsychiatric disturbance due to its long‐acting metabolite.^[^ ^57^, ^58^ ^]^ In contrast to these neuroinhibitory GABA agonists, ketamine, a neuroexcitatory N‐methyl‐D‐aspartate (NMDA) antagonist, possesses both sedative and analgesic properties.^[^ ^59^, ^60^ ^]^ Consequently, ketamine is frequently chosen for procedural sedation, anesthesia induction, postoperative analgesia and pain management.^[^ ^60^, ^61^ ^]^ Perioperative ketamine use typically results in minimal adverse events, primarily encompassing PONV, psychomimetic events, hallucinations, delirium, and hypertension.^[^ ^59^, ^61^ ^]^ However, the repeated use of ketamine for chronic pain management and recreational purposes has been associated with severe hepatobiliary, bladder and urinary tract toxicity.^[^ ^60^, ^62^ ^]^

Since most sedatives lack analgesic properties except for ketamine, opioids with potent analgesic efficacy are the cornerstone of perioperative pain management.^[^ ^63^ ^]^ By activating central and peripheral µ opioid receptors (MORs), opioids inhibit the reflexive and emotional processing of neutron toward pain and exert analgesic effects. However, the indiscriminate stimulation of MORs can lead to various adverse events.^[^ ^7^ ^]^ A systematic review assessing 45 randomized controlled trials revealed that intravenous opioid analgesia was associated with a 75.9% risk of CNS depression (e.g., somnolence, dizziness, psychomotor abnormalities), a 28.2% risk of gastrointestinal symptoms (e.g., constipation, nausea, vomiting), pruritus (incidence rate 17.5%), urinary retention (4.1%), and respiratory suppression (2.4%).^[^ ^64^ ^]^ Furthermore, many surgical procedures are associated with an increased risk of chronic opioid use in the first preoperative year, ranging from 0.119% to 1.41% based on the types of surgery.^[^ ^65^ ^]^ Prolonged administration or misuse of opioids may result in opioid tolerance and hyperalgesia, dependence, withdrawal symptoms, addiction, and, in cases of overdose, fatal outcomes.^[^ ^63^, ^66^ ^]^ Consequently, there is a growing interest in developing novel opioid analgesics with improved safety profiles and exploring alternative approaches to pain management.^[^ ^67^, ^68^ ^]^

Neuromuscular blocking agents (NMBs) are employed to induce muscle paralysis, especially in surgeries involving endotracheal intubation, as motion nerves are less sensitive to anesthetic agents. The sole polarizing NMBs is succinylcholine (Sch) that binds to acetylcholine receptors (AChR) of postsynaptic neurons, inducing hyperpolarization and paralysis. Nonpolarizing NMBs, including pancuronium, rocuronium, atracurium, and cisatracurium competitively antagonize the binding of acetylcholine (ACh) to AChR, thereby blocking neuromuscular transmission.^[^ ^69^ ^]^ Although NMBs improve incubation success rate and reduces airway‐related complications, up to 88% of patients administered NMBs experience residual neuromuscular blockade.^[^ ^70^ ^]^ Impaired function of pharyngeal and airway muscles increases the incidence of respiratory adverse events such as hypoxemia, airway obstruction, postoperative pneumonia, ultimately raising postoperative morbidity and mortality rates.^[^ ^71^ ^]^ Additionally, potential complications related to NMBs include allergic and pseudo‐allergic reactions, and concerns about their impact on cancer recurrence and metastasis.^[^ ^69^ ^]^

### 2.2 Regional Anesthesia, Local Anesthesia, and Related Toxicity

RA serves as a prevalent anesthesia modality and pain relief approach to numb a large part of the body.^[^ ^72^ ^]^ Key RA techniques encompass neuraxial anesthesia and peripheral nerve block. Neuraxial anesthesia involves the administration of local anesthetics and adjuncts into epidural or subarachnoid space, effectively blocking sympathetic conduction to induce anesthesia and analgesia. This technique finds wide application in surgeries involving the abdomen, pelvis, lower extremities, and labor analgesia.^[^ ^73^, ^74^ ^]^ Similarly, peripheral nerve blockades (PNB) inject local anesthetics and additives to the vicinity of peripheral nerves for perioperative anesthesia and analgesia.^[^ ^75^, ^76^ ^]^ Conversely, LA selectively numbs small areas following topical administration or direct infiltration of local anesthetics, which is commonly applied for cutaneous, dental and head and neck procedures.^[^ ^77^, ^78^ ^]^

Despite RA and LA generally exhibiting superior safety compared to general anesthesia,^[^ ^79^, ^80^, ^81^, ^82^ ^]^ the practice is not without risks. Complications may arise due to technical issues, toxicity of anesthetics and additives, or a combination of both factors. Specifically, post‐dural puncture headache is a frequent complication (50%–80%) that arises following dural puncture, which intentionally occurred in subarachnoid anesthesia and inadvertently occurred in epidural anesthesia.^[^ ^83^ ^]^ Bleeding complications occur in ≈1 patient per 20 000 to 168 000 blocks and show extremely low risks in PNB except for deep blocks such as lumbar plexus.^[^ ^84^, ^85^ ^]^ However, patients with coagulopathies or receiving anticoagulation therapies face elevated risks (≈0.67%) of bleeding complications when undergoing PNB. In severe cases, hemorrhage within the epidural or subarachnoid space may lead to cord compression and paraplegia if not promptly addressed.^[^ ^84^, ^86^ ^]^ In addition to bleeding, neurological infections such as epidural abscess, meningitis and arachnoiditis are also extremely rare yet catastrophic complications of neuraxial blockade, which may result in permanent spinal cord injuries.^[^ ^86^, ^87^, ^88^ ^]^ Nerve injuries are other infrequent complications, ≈2–4 in 10 000 for RA and LA, and may result from direct trauma, compression due to high injection pressure or hemorrhage and neurotoxicity induced by chemical agents.^[^ ^77^, ^84^ ^]^

Since local anesthetics are the primary chemicals employed in RA and LA, toxicities associated with local anesthetics are important risks of RA and LA. In neuraxial anesthesia, local anesthetics may frequently elicit hypotension, bradycardia and urinary retention due to their sympatholytic properties.^[^ ^86^ ^]^ In PNB and LA, excessive local anesthetics in system circulation due to inadvertent vascular puncture and/or overdose can lead to mild to severe local anesthetic system toxicity (LAST).^[^ ^89^ ^]^ Characterized by symptoms such as tongue numbness, dizziness, visual disturbances, unconsciousness, seizure, respiratory arrest, cardiovascular depression and even cardiac collapse, Despite the relatively rare (1–11 cases per 10 000 patients) incidence, LAST poses life‐threatening risks once tiggered.^[^ ^8^, ^86^, ^89^ ^]^ Additionally, patients administered ester local anesthetics may experience allergic reactions, while amide local anesthetics have been associated with methemoglobinemia.^[^ ^78^ ^]^

## 3 Nanoformulation of Anesthetics with Good Safety

Many adverse events following anesthesia, such as PONV and cardiovascular depression can be managed with supportive equipment and medications. However, certain anesthesia‐associated complications present challenges that are inadequately addressed by conventional approaches but can significantly benefit from the application of nanotechnology. Among all potential resolutions, the most radical strategy for safe anesthesia practice is to adopt anesthetic formulations with weak side effects. In this context, nano‐formulation of insoluble anesthetics offers a promising avenue to minimize the use of potentially harmful pharmaceutical excipients, such as solubilizers and preservatives, thereby improving the safety profiles of anesthetics. Moreover, nano‐formulation of anesthetics can improve the targeted delivery of anesthetic agents, mitigating the adverse effects associated with off‐target actions of these agents.

**Figure 1:**
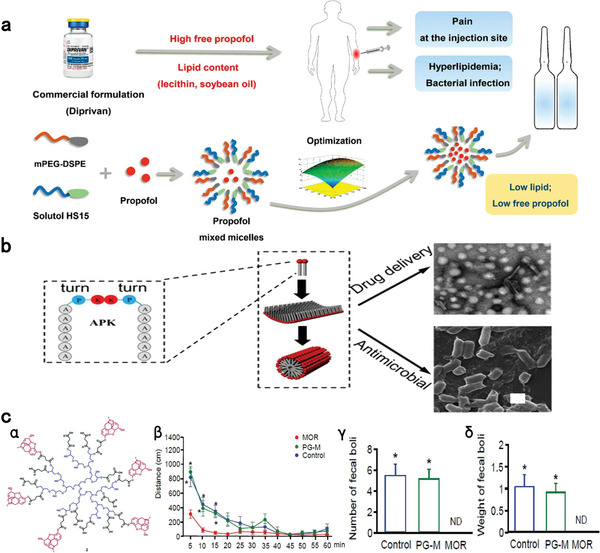
Nano‐formulization for improving the safety of anesthetics. a) Propofol‐loaded mixed micelle exhibited low risks of injection pain, hyperlipidemia and bacterial infection by replacing lipids with co‐polymers. Reprinted with permission.^[^ ^83^ ^]^ Copyright 2022, Chu et al., open access. b) Self‐assembling APK peptides form antibacterial nanofiber that can be used as a safe nano‐formulation for etomidate. Reproduced with permission.^[^ ^87^ ^]^ Copyright 2021, Elsevier publication. c) The structure (α) of peripheral‐limited morphine derivative (PG‐M); (β) Locomotor activity after intravenous injections of morphine (MOR), PG‐M or 0.9% NaCl. ^*^ *p*<0.05, two‐way RM‐ANOVA and Bonferroni test (compared to MOR); Number (γ) and total weight (δ) of fecal boli produced after intravenous injections of MOR, PG‐M or 0.9% NaCl. ND: none detected; ^*^ *p*<0.05, oneway ANOVA and Bonferroni test (compared to MOR); N = 8 rats per group; means ± SEM. Reproduced with permission.^[^ ^88^ ^]^ Copyright 2017, Gonzalez‐Rodriguez et al., open access.

Propofol's high lipophilicity and its irritating alkalinity when forming salt render lipid emulsion an ideal injectable formulation for propofol. Diprivan is a commercially available soybean oil‐based nano‐emulsion for intravenous administration of propofol. Despite its wide application in inducing and maintaining anesthesia, Diprivan suffers from inherent disadvantages attributed to its use of excipients. These drawbacks encompass issues such as injection pain due to the presence of unloaded propofol, the risk of lipid‐induced hyperlipidemia and hypocalcemia with prolonged use, susceptibility to microbial contamination, and instability during long‐term storage.^[^ ^11^, ^90^ ^]^ To tackle these challenges, numerous innovative nano‐formulations have been developed to enhance the efficacy and safety of propofol, while avoiding the use of soybean oil and disodium edetate.^[^ ^11^, ^90^, ^91^ ^]^ For example, Shevalkar et al. devised a nanostructured lipid carrier (NLC) of propofol using the hot emulsification‐probe sonication method and refined the combinations of solid lipids, oils and surfactants. This optimized NLC formulation not only reduced the content of free propofol in the aqueous phase by 40%, thereby alleviating injection pain compared to Diprivan, but also exhibited other favorable attributes such as non‐hemolysis, comparable pharmacokinetics, reduced risk of hyperlipidemia, improved long‐term storage stability, and antimicrobial properties.^[^ ^11^ ^]^ Furthermore, low‐lipid and lipid‐free nanostructures have garnered considerable interest due to their diminished hyperlipidemia risk.^[^ ^92^, ^93^ ^]^ Reportedly, amphipathic copolymer distearoyl‐phosphatidylethanolamine‐methoxy‐poly‐(ethylene glycol 2000) (DSPE mPEG2k) and solubilizer Solutol HS15 were used to fabricate micelles by a film dispersion method, which was proved to be an improved formulation for propofol.^[^ ^92^ ^]^ The optimized propofol micelle displayed a 60.9% lower free propofol concentration than that of Diprivan, leading to a greatly alleviated pain symptom (**Figure** **1**a). Qiu et al. developed a self‐assembling peptide (SAP) nanoparticle based on the GQY peptide with an amino acid sequence of GQQQQQY for propofol encapsulation. Propofol‐GYQ was fabricated by dissolving GYQ and propofol into 5% glucose injection under sonification and magnetic stirring followed by incubation for 24 h. Propofol‐GYQ exhibited high encapsulation of propofol (96.59%), minimal painful irritation at the injection site, no support for microbial growth, comparable anesthetic efficacy to Diprivan, and good biocompatibility.^[^ ^93^ ^]^

Similar to propofol, etomidate is a highly hydrophobic drug frequently used in routine anesthesia. To achieve the necessary concentration for effectiveness, current commercially available etomidate formulations utilize either lipid emulsion (e.g., Etomidate‐Lipuro) or propylene glycol (e.g., Amidate) as solubilizing agents.^[^ ^11^ ^]^ However, these solubilizing agents have been associated with various adverse events, including propylene glycol‐related transient venous pain, acidosis, seizures, hemolysis, acute tubular necrosis, soybean‐induced hyperlipidemia and allergic reactions.^[^ ^9^, ^52^, ^94^ ^]^ Hence, there is a pressing need to develop novel etomidate formulations that circumvent the use of adverse excipients, particularly propylene glycol. In this context, lipid nano‐emulsions, micelles and SAP‐based nanoplatforms have been explored to create etomidate preparations with inherently improved safety profiles.^[^ ^9^, ^95^, ^96^ ^]^ For instance, Geng et al. developed a lipid emulsion using a high‐pressure homogenization method for the parenteral delivery of etomidate termed ETM‐ILE. ETM‐ILE presented good long‐term stability at room temperature, negligible hemolytic and venous irritation effects, PK features similar to Amidate, and a slightly different distribution pattern.^[^ ^95^ ^]^ Further research is warranted to evaluate ETM‐ILE's anesthetic efficacy and potential superiority over Etomidate‐Lipuro. Although ETM‐ILE eliminates the risk of propylene glycol, high lipid content introduces inherent safety concerns that may be addressed through lipid‐free formulations. For instance, an etomidate‐loaded mixed micelle, denoted as Eto‐PFM, was developed based on Pluronic P123 and F108 by a thin‐film hydration method. Eto‐PFM presented strong encapsulation of etomidate, thereby avoiding the inclusion of soybean oil.^[^ ^9^ ^]^ In comparison to the marketed etomidate emulsion, Eto‐PFM displayed rapid drug release kinetics, similar anesthetic effects and safety.^[^ ^9^ ^]^ Furthermore, Qiu et al. utilized a surfactant‐like peptide denoted as APK with a feature of a turn conformation and two tails to form self‐assembling nanoparticles, nanosheets and/or nanofibers for delivering various hydrophobic drugs, including etomidate. The preparation process of etomidate‐encapsulated APK nanostructure is facile and involves suspension of etomidate into APK solution under vigorous stir and subsequent ultrasound treatment. The cationic amphiphilic APK and its nanofiber structure endowed it with self‐antimicrobial capability by disrupting microbial membranes and inhibiting microbial movement and proliferation. Etomidate‐loaded APK exhibited remarkable safety, comparable anesthetic performance to its clinical counterpart and significantly stronger self‐antimicrobial activity (Figure 1b).^[^ ^96^ ^]^

A significant proportion of complications associated with opioid use, such as sedation, apnea, and addiction, can be attributed to the inappropriate activation of CNS opioid receptors. Achieving targeted delivery of opioid analgesics to peripheral injury or inflammatory sites offers the potential for efficient analgesia without CNS‐related side effects.^[^ ^97^, ^98^ ^]^ To this end, morphine was covalently conjugated with hyperbranched polyglycerol through a cleavable linker, resulting in PG‐M. PG‐M, characterized by its high molecular weight and hydrophilicity, was designed to prevent its penetration through the blood–brain‐barrier (BBB). Intravenous injection of PG‐M was shown to selectively release morphine in the peripheral inflamed environment, producing analgesia without central and intestinal toxicity, as evidenced by increased locomotor activity and bowel movements (Figure 1c).^[^ ^97^ ^]^ Inspired by this attempt, other strategies for achieving peripheral‐limited and inflamed site‐targeting opioid formulations could be explored by modifying drug administration routes and adjusting the physicochemical properties of nanoplatforms such as size and surface modifications. For instance, loperamide‐encapsulated liposomes were conjugated with antibodies against intercellular adhesion molecule‐1 (anti‐ICAM‐1) for pain management at peripheral inflamed sites.^[^ ^99^ ^]^ The immunoliposome was constructed by incubating thiolated antibody with conventional liposomes made using a dried lipid film hydration method. This immunoliposome exerted potent anti‐inflammatory and analgesic effects without inducing CNS‐related complications. It is important to note that further investigation is required to thoroughly assess the efficacy and safety of these peripheral‐limited opioid formulations. Additionally, other strategies for achieving safe opioid analgesia, such as developing κ‐receptor biased ligands, heteromers bivalent and multifunctional ligands, and µ‐receptor splice variants, are beyond the scope of this nanotechnology‐focused discussion and can be found in relevant reviews.^[^ ^98^, ^100^, ^101^ ^]^

## 4 PK Approaches: Nano‐Antidotes Sequestering Anesthetics for Detoxication

Apart from developing safe nano‐formulations for anesthetics, using nano‐antidotes to sequester or inactivate overdosed poisonous drugs is an effective detoxification approach. Unlike drugs with pharmacological activities for mitigating disease symptoms, nano‐antidotes are engineered to capture or sometimes metabolize overdosed offending xenobiotics, resulting in decreased free concentrations of toxic xenobiotics and the redistribution of xenobiotics from the site of toxicity.^[^ ^13^ ^]^ According to their mechanisms of action, nano‐antidotes can be divided into three categories: general nano‐antidotes relying on nonspecific interactions to sequester xenobiotics (e.g., electrostatic and/or hydrophobic interactions), specific nano‐antidotes capturing xenobiotics via specific chemical bonding, and catalytic nano‐antidotes with biomimetic metabolizing capability to metabolize/inactivate xenobiotics.^[^ ^13^, ^102^, ^103^ ^]^ In the field of anesthesia, overdoses of anesthetics, such as propofol, opioids and local anesthetics can cause devastating systemic toxicity, including cardiorespiratory arrest and CNS dysfunction. However, current management for these life‐threatening complications is limited to supportive measures, which could potentially be complemented by effective nano‐antidotes to collectively reduce morbidity and mortality.^[^ ^13^, ^103^ ^]^ It should be noted that few nano‐antidotes may simultaneously possess PK‐altering capability and pharmacological activities. The focus of this section is primarily on current and prospective nano‐antidotes that detoxify overdosed anesthetics by altering their PK process.

### 4.1 Lipid Emulsion, Anionic Liposome/Nanoparticle, and pH‐Gradient Liposome

**Figure 2:**
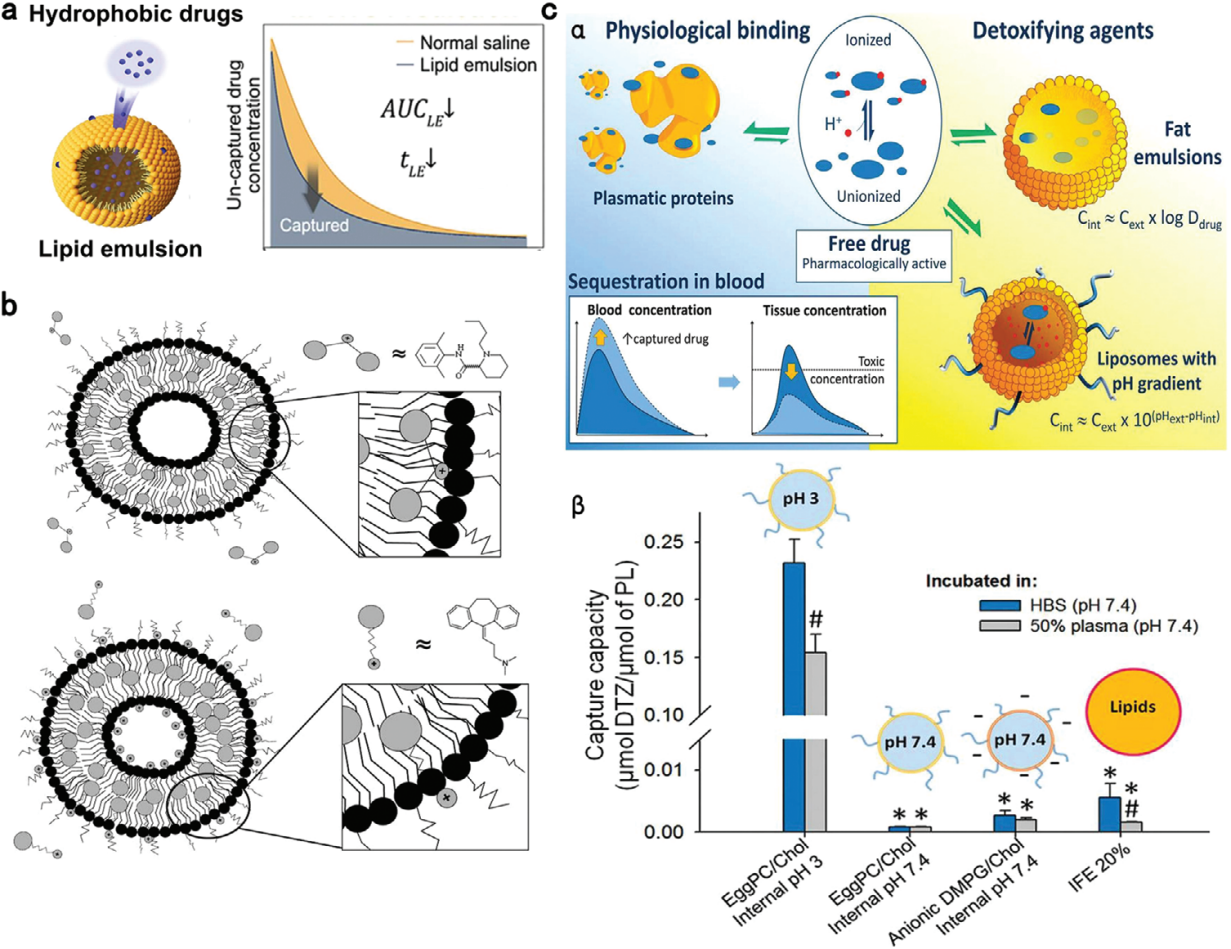
Schematic illustration of lipid‐based nano‐antidotes for counteracting drug overdose. a) Detoxifying mechanisms of lipid emulsions based on ‘lipid‐sink’ theory. Reproduced with permission.^[^ ^92^ ^]^ Copyright 2022, Elsevier publication. b) Potential interactions of anionic liposomes with cationic drugs. Reproduced with permission.^[^ ^108^ ^]^ Copyright 2009, American Chemical Society (ACS) publication. c) The rationale of using transmembrane pH‐gradient liposomes as antidotes (α); (β) In vitro capturing capacity of various nanoformulations toward diltiazem in 50% plasma and HEPES‐buffered saline (HBS). Values represent means ± SD, n = 3. Values are normalized for the number of phospholipids in the formulation; ^#^ *p* < 0.05 versus the same formulation in HBS, ^*^ *p* < 0.05 versus liposomes with internal pH 3 in plasma. Reproduced with permission.^[^ ^110^ ^]^ Copyright 2010, ACS publication.

An typical broad‐spectrum nano‐antidote, especially for lipophilic drug overdose, is intravenous lipid emulsion (e.g., Intralipid), which is a nanosized oil‐in‐water emulsion.^[^ ^102^ ^]^ Since the successful rescue of a patient from bupivacaine‐induced cardiac arrest with Intralipid in 2006, lipid resuscitation therapy has been adopted to mitigate LAST in emergency.^[^ ^102^, ^104^ ^]^ Notably, Intralipid was approved by FDA as a nutrition supplementary to provide energy and prevent essential fatty acid deficiency. LAST is still not an official indication of Intralipid. Although not fully understood, its detoxifying mechanisms are believed to rely predominantly on the ‘lipid sink’ and ‘lipid shuttle’ theory in which lipid droplets sequester the overdosed drug and transfer it to less vulnerable organs (**Figure** **2**a). Other detoxifying effects mainly stem from the bioactivity of lipids, including enhanced energy provision and cardiotonic and vasoconstrictive abilities.^[^ ^13^, ^105^, ^106^, ^107^ ^]^ Based on the lipid sequestration and redistribution mechanism, several preclinical studies and case reports have suggested the application of lipid emulsion to detoxify other hydrophilic anesthetic drugs such as propofol, cocaine and tramadol.^[^ ^108^, ^109^, ^110^ ^]^

However, there are still discrepancies regarding the effectiveness and widespread application of lipid rescue therapy.^[^ ^111^ ^]^ Concerns about the lack of randomized and controlled clinical trials, heterogeneous efficacy, publication bias of underreported treatment failure, toxicity relapse of overdosed drugs and lipid‐related complications have hindered the official approval of lipid emulsion for any antidotal therapy.^[^ ^13^, ^111^, ^112^, ^113^ ^]^ Specifically, toxicity recurrence may result from both the redistribution of lipid droplets to the well‐perfused heart and brain and the reversible sequestration of drugs by lipid emulsion.^[^ ^13^, ^114^ ^]^ Furthermore, when administered in large doses, intravenous lipid emulsion can lead to various adverse events, including pyrogenic reactions, fat embolism, respiratory injury, pancreatitis, hypertriglyceridemia, etc.^[^ ^113^ ^]^ Evaluating the trade‐offs of lipid rescue therapy, carefully selecting the dose and dose rate of lipid emulsion, and modulating the particle size and lipid component may improve its antidotal performance and safety profiles.

In addition to lipid emulsion, anionic nano‐antidotes based on liposomes or polymeric nanoparticles have been proven to sequester weak base drugs such as bupivacaine (pKa 8.1) and amitriptyline (pKa 9.4), which primarily exist in cationic forms in the physiological environment.^[^ ^115^, ^116^, ^117^ ^]^ Increasing the proportion of acidic functional groups can enhance electrostatic interactions between anionic nano‐antidotes and cationic drugs, resulting in tighter capture. Additionally, hydrophobic interactions between the uncharged part of drugs and the lipophilic portion of nano‐antidotes also contribute to this process (Figure 2b).^[^ ^118^ ^]^ The anionic liposomes can be prepared by common film hydration/extrusion method with increased component ratio of anionic lipid, for example, 1,2‐dioleoyl‐sn‐glycero‐3‐[phospho‐rac‐(1‐glycerol)] (sodium salt) (DOPG).^[^ ^118^ ^]^ Polyethylene glycol (PEG) functionalization not only inhibits protein adsorption onto the surface of nano‐antidotes to improve drug binding but also prolongs nano‐antidotes’ circulation time, further reducing toxicity.^[^ ^12^, ^119^ ^]^ Similar to lipid emulsion, anionic nano‐antidotes also provide reversible sequestration, which may lead to drug leakage and even toxicity recurrence. To this end, transmembrane pH‐gradient liposomes with acidic interior (pH 3–4) provide an irreversible drug‐trapping method. Liposomes with acidic aqueous cores are particularly suitable for amphiphilic weak bases, which are lipophilic in the blood to penetrate through lipid bilayer and then are protonated and confined in the acidic interior.^[^ ^12^, ^103^ ^]^ For example, Leroux et al. fabricated a series of PEGylated liposomes with different inner pH and surface charge and studied their capturing and detoxifying capabilities with diltiazem (pKa 8.18, LogP 2.8) in vitro and in vivo (Figure 2c).^[^ ^120^ ^]^ The film hydration/extrusion method was adopted for liposome fabrication and the difference in inner pH was achieved by changing the buffer solution for hydration. Liposomes with an internal pH 3 were hydrated in citrate buffer and exhibited a 40‐fold stronger trapping ability in the presence of serum than that of Intralipid and the anionic liposomes with a pH 7.4. Neutral liposomes with an internal pH 7.4 showed the weakest diltiazem‐trapping performance. In vivo studies revealed that pH‐gradient liposomes limited diltiazem's tissue distribution and moderated its overdosed toxicity. Similar acidic center‐liposomes were developed for detoxication of other drugs such as verapamil (pKa 8.92, LogP 3.79).^[^ ^121^, ^122^ ^]^ To the best of our knowledge, no such pH gradient liposomes have been developed for mitigating the intoxication of anesthetic drugs. Based on the ionization and partition profiles, some local anesthetics like bupivacaine with a pKa of 8.1 and LogP of 3.41 might be feasible targets for pH‐gradient liposome antidotes,^[^ ^12^ ^]^ which requires further experimental research.

### 4.2 (Nano)‐Antidotes with High Affinity Recognizing Units

To further enhance detoxification efficiency, high‐affinity nano‐antidotes based on specific recognition of particular drugs or drug families are highly desirable. Their targeted drug‐capturing capability can stem from antigen‐antibody interactions, ligand‐receptor binding, enzymatic‐substrate recognition, and guest‐host complexation using antibodies, customized molecularly imprinted polymers (MIPs), DNA devices, aptamers, and supramolecules, among others.^[^ ^13^, ^103^ ^]^

**Figure 3:**
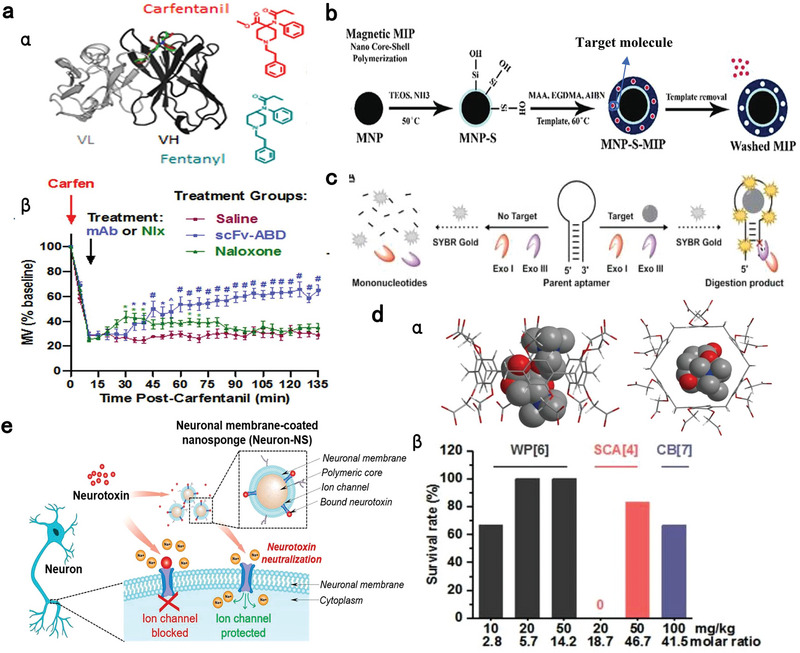
Potential (nano)‐antidotes specifically sequestering anesthetics for detoxification. a) Structure (α) of humanized antibodies against fentanyl and its analogs. (β) Reversal of carfentanil‐induced respiratory depression by the antibody and naloxone. n = 8 per group. Data presents as means ± SEM. Two‐way RM ANOVA was employed for statistical analysis; ^*^ *p* > 0.05, ^#^ *p* > 0.001. Reproduced with permission.^[^ ^115^ ^]^ Copyright 2023, ACS publication. b) Scheme of MIPs‐coated magnetic nanocore‐shell structure with high extraction efficiency of templates. Reproduced with permission.^[^ ^123^ ^]^ Copyright 2022, Elsevier publication. c) Schematic illustration of a label‐free exonuclease digestion assay for developing aptamers with high affinity to fentanyl. Reproduced with permission.^[^ ^128^ ^]^ Copyright 2022, Canoura et al., open access. d) Schematic illustration (α) of energy‐optimized inclusion complexes of WP[6] and Sch; (β) Survival rates of mice intravenously treated with various doses of synthetic receptors immediately after the administration of a lethal dose of Sch (0.75 mg kg^−1^). n = 6 for each group. Reproduced with permission.^[^ ^132^ ^]^ Copyright 2019, Zhang et al., open access. e) Neuronal membrane‐covered nanosponges serve as neuron decoys to sequester neurotoxins. Reproduced with permission.^[^ ^117^ ^]^ Copyright 2022, ACS publication.

Antibody‐based detoxifiers enable more precise yet narrower‐spectrum capture of targeted drugs or antigens. Eubanks et al. developed a human monoclonal anti‐cocaine antibody (GNCazk) and its clinically relevant recombinant IgG form, revealed their exceptional specificity and rapid association with cocaine, which ensure their efficient protection against cocaine overdose.^[^ ^123^ ^]^ Murine, chimeric and humanized antibodies have been developed to capture or catalyze fentanyl and its analogs to counteract opioid toxicity.^[^ ^124^, ^125^, ^126^ ^]^ For instance, a humanized monoclonal antibody, C10‐S66K displayed a high binding capacity to fentanyl and carfentanil (100‐fold more potent than fentanyl). Intraperitoneal injection of 30 mg kg^−1^ C10‐S66K antibody significantly alleviated respiratory depression caused by carfentanil overdose (**Figure** **3**a).^[^ ^125^ ^]^ Nevertheless, antibody‐based antidotal therapy may suffer from high production costs, large doses required for detoxification, a narrow detoxifying spectrum, potential immunogenicity and low stability.^[^ ^13^, ^127^ ^]^ In addition to antibody‐based passive immunization, active vaccination via opioid‐conjugated vaccines might be an effective alternative therapy for the opioid crisis. Matyas et al. developed a bivalent vaccine composed of heroin hapten‐ and fentanyl hapten‐conjugated tetanus toxoid with liposomes and aluminum hydroxide as immunologic adjuvants for treating opioid overdose.^[^ ^128^ ^]^ However, it's worth noting that active vaccination may not be suitable for addressing opioid overdose in the field of anesthesiology, as it could negate opioids’ analgesic effects, which are highly relied upon for perioperative pain management. Another reason is that active vaccination takes a considerable amount of time to establish immunity and is unlikely to address sudden opioid overdoses.

Another platform with exquisite recognizing ability comparable to that of antibodies is MIPs, which may shed light on the development of large‐scale, cost‐effective and high‐affinity nano‐antidotes.^[^ ^13^ ^]^ The fabrication of MIPs involves the copolymerization of functional monomers and cross‐linkers around target templates, followed by the removal of target templates.^[^ ^129^ ^]^ Theoretically, all sorts of small molecules and biomacromolecules can serve as templates to produce their complementary MIPs, which specifically recognize these templates based on spatial topology and covalent and noncovalent interactions.^[^ ^129^ ^]^ By merits of MIPs’ robust recognition specificity, good stability, ease of scale‐up, tunable synthesis and favorable reusability, MIPs have become a promising modality in the biomedical field for applications such as sample separation and purification, biosensing, disease diagnosis and therapeutics, etc.^[^ ^129^, ^130^ ^]^ Although no study has been conducted to alleviate anesthetics‐related toxicity using MIPs, MIPs have exhibited appreciable potential in sequestering anesthetics for antidotal therapy. Polyvinyl alcohol‐based molecularly imprinted microspheres were reported to act as the solid‐phase extraction materials to extract benzocaine and resulted in over a 90% recovery rate of benzocaine in human serum and fish tissues.^[^ ^131^ ^]^ To overcome the deficiencies of bulk or micro/nanoparticle MIPs, such as incomplete template removal, slow mass transfer and low binding rate, MIPs‐based surface coating on nanoplatforms could achieve higher sequestering capacity and efficiency for the target template (Figure 3b).^[^ ^132^, ^133^ ^]^ For example, magnetic silica‐coated MIPs (MNP‐SMIPs) for extracting amiodarone were developed by evenly dispersing MNP‐S NPs into mixtures containing amiodarone (template), MAA (polymer monomer) and EGDMA (crosslinker) for thermal polymerization, which was followed by ultrasound extraction to remove the template and magnet extraction of the final MNP‐SMIPs. The maximum adsorption capacity of MNP‐SMIPs for amiodarone was ≈15‐fold higher than that of the bulk MIPs.^[^ ^133^ ^]^ MIPs‐facilitated nano‐antidotes present some feasibility for antidotal therapy, and further cellular and animal experiments should be conducted to validate their detoxifying effectiveness.

Similar to antibodies and MIPs, aptamers are artificial single‐strand oligonucleotide chains with high affinity for specific targets, which encompass proteins, metal ions, organic chemicals, cells and viruses, etc.^[^ ^134^ ^]^ Driven by the propensity to form complementary base pairs, aptamers with different sequences can fold into diverse secondary structures (e.g., stem, loop and G‐quadruplex), which further transform into unique 3D structures for target recognition via shape complementarity, hydrogen bonding, hydrophobic and electrostatic interactions, and base accumulation.^[^ ^135^ ^]^ With the advent and improvement of systematic evolution of ligands by exponential enrichment (SELEX) technology, customized aptamers with high affinity and specificity to targets can be fabricated in a rapid, cost‐effective and quality‐steady manner, greatly fostering aptamers’ applications in biosensing, disease diagnosis and targeted therapeutics.^[^ ^135^, ^136^, ^137^ ^]^ Despite current preliminary investigations, aptamer‐based antidotes represent a promising therapy for anesthetics intoxication. For instance, Xiao et al. developed a high‐throughput method to accurately identify aptamers against fentanyl and its analogs. SELEX was used to generate 28 aptamer candidates, which were further screened through the exonuclease digestion assay and finally generated the optimist aptamers (Figure 3c). An aptamer denoted as F27 showed a high affinity for fentanyl and can detect fentanyl at concentrations as low as 15 nM in complex biological matrices.^[^ ^138^ ^]^ However, in vivo applications of unmodified or unformulated aptamers may suffer from fast kidney filtration, susceptible nuclease degradation and a short circulation time, which could be overcome by integrating with nanoplatforms.^[^ ^13^, ^135^ ^]^ Wang et al. developed a long‐tentacle jellyfish‐like open nano‐antidote system, denoted as PA‐NPs for efficient and rapid sequestration of thrombin. Specifically, the sequence of the DNA inhibitor (DI) and DNA monomer (DM) was designed using the NUPACK server. Streptavidin‐coated iron oxide NPs act as the molecular support for the biotinylated DI‐DM‐based long‐chain DNA polymers with overhangs, which further hybridize with aptamers against thrombin. The resulted PA‐NPs sequestered 70% of thrombin from the solution and greatly prolonged the coagulation time.^[^ ^139^ ^]^ In theory, this nanoplatform can be extrapolated to trap any target molecules with high‐affinity aptamers. For instance, the integration of F27 may yield an efficient nano‐antidote against fentanyl, but this requires in‐depth in vivo research for validation.

In addition to the aforementioned units with high recognizing ability, versatile supramolecules have been identified to form sufficiently tight guest‐host complexes with anesthetic drugs, including neuromuscular blockers, opioids, local anesthetics, ketamine and etomidate.^[^ ^140^, ^141^ ^]^ These supramolecule hosts, also known as artificial receptors, include cyclodextrins (CD), cucurbiturils (CB[n]), pillararenes (PA[n]), and sulfo‐calixarenes (SCA[n]), which typically possess a hydrophobic cavity and a hydrophilic exterior to capture organic guests. Remarkably, Sugammadex, a γ‐CD derivative, has been successfully approved in over 50 countries for reversing the residual muscular‐relaxing effects of non‐depolarizing steroidal NMBAs.^[^ ^142^ ^]^ Inspired by Sugammadex, various CDs, CB[n], PA[n] and frameworks have been investigated and showed different binding affinities and in vivo detoxication to NMBAs.^[^ ^140^, ^142^, ^143^ ^]^ For example, carboxylatopillar[6]arene (WP[6]) presented the highest affinity for Sch with a binding constant of 2.79 × 10^5^ in the PBS solution, which is higher than that of CB7, SCA4, ACh, AChR, and choline (potential competitive guests). WP[6] injection immediately following Sch overdose improved survival rate, reversed paralysis and alleviated cardiac arrhythmia, hyperkalemia, and muscular damage (Figure 3d).^[^ ^142^ ^]^ Supramolecules have also been employed for treating opioid intoxication. Calabadion1, a representative of CB[n], was found to efficiently sequester fentanyl in the circulatory system to counteract respiratory depression, CNS dysfunction and muscle rigidity in a rat model.^[^ ^144^ ^]^ Isaacs et al. revealed that Pillar[6]MaxQ (P6AS) forms tight complexation with fentanyl and can reverse 0.1 mg kg^−1^ fentanyl‐induced hyperlocomotion when administered 15 min after fentanyl administration, which is promising for practical fentanyl detoxication.^[^ ^145^ ^]^ As for detoxifying supramolecules for local anesthetics, CDs, CBs and SCAs exhibited relatively low affinities and required further modification for effective antidotal therapy.^[^ ^146^ ^]^ Furthermore, integrating supramolecules with nanomaterials may have synergistic effects on antidotal therapy, which is also an interesting field to investigate.

### 4.3 Biomimetic Nanosponges as Efficient Nano‐Antidotes

In contrast to the narrow trapping spectrum of high‐affinity recognizers, nanosponges, as bioactive membrane‐coated nanodevices, show promise as antidotal nanoplatforms for certain xenobiotic families, where members have similar toxicological targets.^[^ ^127^, ^147^ ^]^ Various cellular membranes originating from different kinds of cell lines have been integrated with nanoplatforms to form host decoys for hazardous xenobiotics.^[^ ^148^ ^]^ Typically, nanosponges made from membranes of red blood cells or platelets are optimal decoys for trapping bacterial toxins and organophosphates (OPs); macrophage and neutrophil membrane‐coated nanosponges have high affinities for inflammatory cytokines, making them suitable to treat immune disorders. When properly covered with host cell membranes, nanosponges could serve as potent countermeasures against viral infections.^[^ ^148^ ^]^ The key to developing robust detoxifying nanosponges lies in the choice of bioactive membranes, which can be cellular/organelle membranes or artificial membranes with specific receptors to strongly interact with target xenobiotics.

The most widely used approach for manufacturing biomimetic nanosponges is to coat purified biomembranes onto the surface of NPs.^[^ ^147^, ^149^ ^]^ Cell membranes from specific sources can be obtained by hypotonic lysing of the respective cells. The biomembrane ghost can then be co‐extruded with preformed NPs through porous membranes to form a core‐shell nanostructure. In addition to physical extrusion, sonication‐based method can also disrupt and reconstruct the biomembrane onto the surface of preformed NPs, resulting in biomimetic nanosponges with the core‐shell feature.^[^ ^127^, ^149^ ^]^

Properly designed nanosponges are highly desirable for treating anesthetics overdose and related complications. Notably, a biomimetic nanosponge denoted NarcroBond was reported to efficiently sequester a comprehensive spectrum of opioids through its multifunctional surface. NarcroBond features a biomimetic lipid bilayer membrane, which is functionalized with recombinant µ receptors to capture opioids and other proteins extracted from neuron membranes to facilitate opioid receptor functions. Additionally, erythrocyte membranes are included to achieve long blood circulation.^[^ ^150^ ^]^ Animal studies demonstrated that NarcroBond counteracted fentanyl‐induced antinociception, respiratory depression and bradycardia by sequestering over 70% of fentanyl in the blood, which was eventually metabolized by the liver along with NarcroBond.^[^ ^151^ ^]^ Importantly, NarcroBond did not interfere with the antagonizing performance of Naloxone, making it a promising candidate for treating the opioid crisis.

Given that most anesthetic drugs (e.g., local anesthetics, sedatives, opioids, and muscular relaxants) exert their pharmacological or toxicological effects by acting on ion channels and/or receptors on neuron membranes, neuromembrane‐coated nanosponges hold great potential to counteract anesthetics intoxication. Inspired by this notion, Zhang et al. devised neuronal membrane‐coated nanosponges (Neuron‐NS), which acted as neuron decoys to capture neurotoxins and exhibited effective therapeutic and prophylactic detoxication for tetrodotoxin overdose (a selective sodium channel blocker) in a mouse model (Figure 3e).^[^ ^127^ ^]^ While anesthetics were not the testing models for Neuron‐NS, it is reasonable to assume that anesthetics could be potential targets sequestered by neuromembrane‐coated nanosponges, pending further experimental validation. In addition to engineering the natural or artificial surface wrapping of nanosponges, a well‐designed inner core is believed to improve the toxin‐sequestering ability for multi‐module detoxification. For example, Zhang et al. reported a dual‐modal biomimetic nanosponge by integrating red blood cell membranes with oil nanodroplets (“Oil‐NS”) for OPs neutralization. The detoxifying mechanism of Oil‐NS involves: 1) acetylcholinesterase (AChE) on the red blood cell membrane binding with OPs through inherent biological recognition; 2) the oil core absorbing lipophilic OPs via physical partition. In therapeutic and prophylactic regimens, Oil‐NS reduced the activity loss of natural AChE, mitigated the hazardous effects of three OPs with distinct structures, and significantly improved the survival rate of mice.^[^ ^152^ ^]^

## 5 PD Approaches: Nanomedicines for Treating Anesthesia‐Related Complications

In addition to sequestering overdosed anesthetics and altering their PK profiles for detoxification, developing therapeutic nanomedicine with intrinsic bioactivities and/or based on pharmaceutical payloads is another effective countermeasure to alleviate anesthesia‐related toxicity. Various nanomaterials have been extensively investigated for their inherent bioactivities, owing to their unique structural and physicochemical properties. The representative nanomaterials with innate potent reactive oxygen species (ROS) scavenging capability include, but are not limited to gold NPs, ceria NPs, selenium NPs, graphene, fullerenol, and various nanozymes, which combat diseases by eliciting antioxidative, anti‐apoptotic and anti‐inflammatory effects.^[^ ^153^, ^154^, ^155^ ^]^ Moreover, certain nanomaterials can directly facilitate the removal and degradation of pathogenic substances, such as Aβ plaque and tau proteins to ameliorate cognitive deficits.^[^ ^156^, ^157^ ^]^ Beyond their intrinsic bioactivities, nanomaterials also serve as intelligent drug delivery systems, enhancing not only the solubility, bioavailability, and stability of drugs but also enabling long‐term circulation, sustained/rapid/stimuli‐responsive release and targeted delivery. This section is dedicated to illustrating how nanomedicine can be harnessed to counteract anesthesia complications from a PD perspective.

### 5.1 Anesthetics‐Related Neurological Disorders

Surgery, anesthesia and the administration of perioperative medications can induce adverse effects on CNS, giving rise to conditions such as POCD, epileptic seizures, acute opioid crises and chronic opioid addiction.^[^ ^7^, ^158^, ^159^ ^]^ POCD, a prevalent anesthesia‐associated complication, is often self‐limiting, yet it can have severe consequences in vulnerable elderly individuals. Currently, there is no specific medication available for the treatment of POCD. Other neurological complications can be managed through corresponding symptomatic treatments, such as antiepileptic drugs and opioid antagonists. However, conventional therapeutics for CNS diseases often yield unsatisfactory efficacy due to the presence of BBB, which impedes the entry of 98% of small molecules and nearly all macromolecules.^[^ ^160^ ^]^ To address this challenge, targeted CNS drug delivery enabled by nanotechnology offers a promising avenue to effectively treat anesthesia‐related CNS toxicity.

#### 5.1.1 Strategies for Overcoming the BBB

Comprehending the structural and physiological characteristics of the BBB is a prerequisite for developing brain‐targeted nanoplatforms. The BBB represents a formidable physical and biochemical barrier that separates the bloodstream from the brain. Its integrity is vital for maintaining normal physiological processes and safeguarding the brain from potentially harmful insults.^[^ ^160^ ^]^ The inner layer of the BBB consists of brain capillary endothelial cells, tightly cohered by tight junctions (TJs) and adherens junctions (AJs), with a physiological width of 1.4–1.8 nm. These cells are equipped with various receptors and transporters with apicobasal polarity, which facilitate the transportation of essential nutrients and waste products.^[^ ^161^ ^]^ Notably, based on the category of transporters/receptors and substances, endocytosis or transcytosis may occur, leading to endothelial lysosome sequestration or endothelial cell penetration, respectively. Particularly, transcytosis is more favorable for brain‐targeted drug delivery.^[^ ^161^ ^]^ The middle layer of the BBB, known as the basement membrane, comprises peripheral cells and the matrix, whereas astrocytes and extracellular matrix form the outer layer of the BBB.^[^ ^160^ ^]^ Besides the physical barrier, various enzymes located inside or on cell membranes (e.g., cytochromes P450 and peptidases) pose an enzymatic challenge to foreign molecules attempting to enter the brain.^[^ ^161^ ^]^ The intricate interplay among endothelial cells, peripheral cells, astrocytes, and microglia (e.g., macrophages) dynamically regulates the BBB permeability.

Nanoplatform‐mediated strategies for bypassing or penetrating the BBB have undergone extensive investigation as potential approaches for efficiently treating various neurodegenerative diseases, seizures, stroke and traumatic brain injuries.^[^ ^156^, ^160^, ^162^ ^]^ Regardless of the limited applications in anesthesia‐related CNS complications, these brain‐targeting strategies can be applicable and valuable. Here, a brief and summative illustration of these BBB‐crossing strategies is provided and in‐depth demonstration can be found in these reviews.^[^ ^14^, ^160^, ^161^, ^163^, ^164^, ^165^ ^]^

Transiently widening the intercellular TJs/AJs between brain capillary endothelial cells has been achieved by providing biochemical stimuli (e.g., hyperosmotic solutions, pharmaceutical molecules) and physical stimuli (e.g., magnetic resonance‐guided focused ultrasound (MRgFUS), electromagnetic field (EMF) and photo stimulation).^[^ ^160^, ^161^ ^]^ Dating back to the 1970s, intra‐arterial injection of hyperosmotic mannitol was demonstrated to induce BBB endothelial dehydration and shrinkage, resulting in temporary TJ opening.^[^ ^166^ ^]^ This pioneering discovery has translated into the use of intra‐arterial mannitol injection as a frontline therapy for cerebral edema and as a preclinical or clinical approach to enhance BBB permeability before administering therapeutic agents for CNS disorders.^[^ ^167^, ^168^ ^]^ Other hyperosmotic solutions with osmotic BBB‐opening ability include fructose, glycerol, acetamide, urea, etc.^[^ ^160^, ^166^ ^]^ However, osmotic BBB opening exhibits high individual variability and carries inherent risks of neurotoxicity.^[^ ^169^ ^]^ For example, improper infusion of mannitol solution at high rates may lead to direct brain injury, which can likely be overcome by monitoring local perfusion with real‐time MRI.^[^ ^167^ ^]^

**Figure 4:**
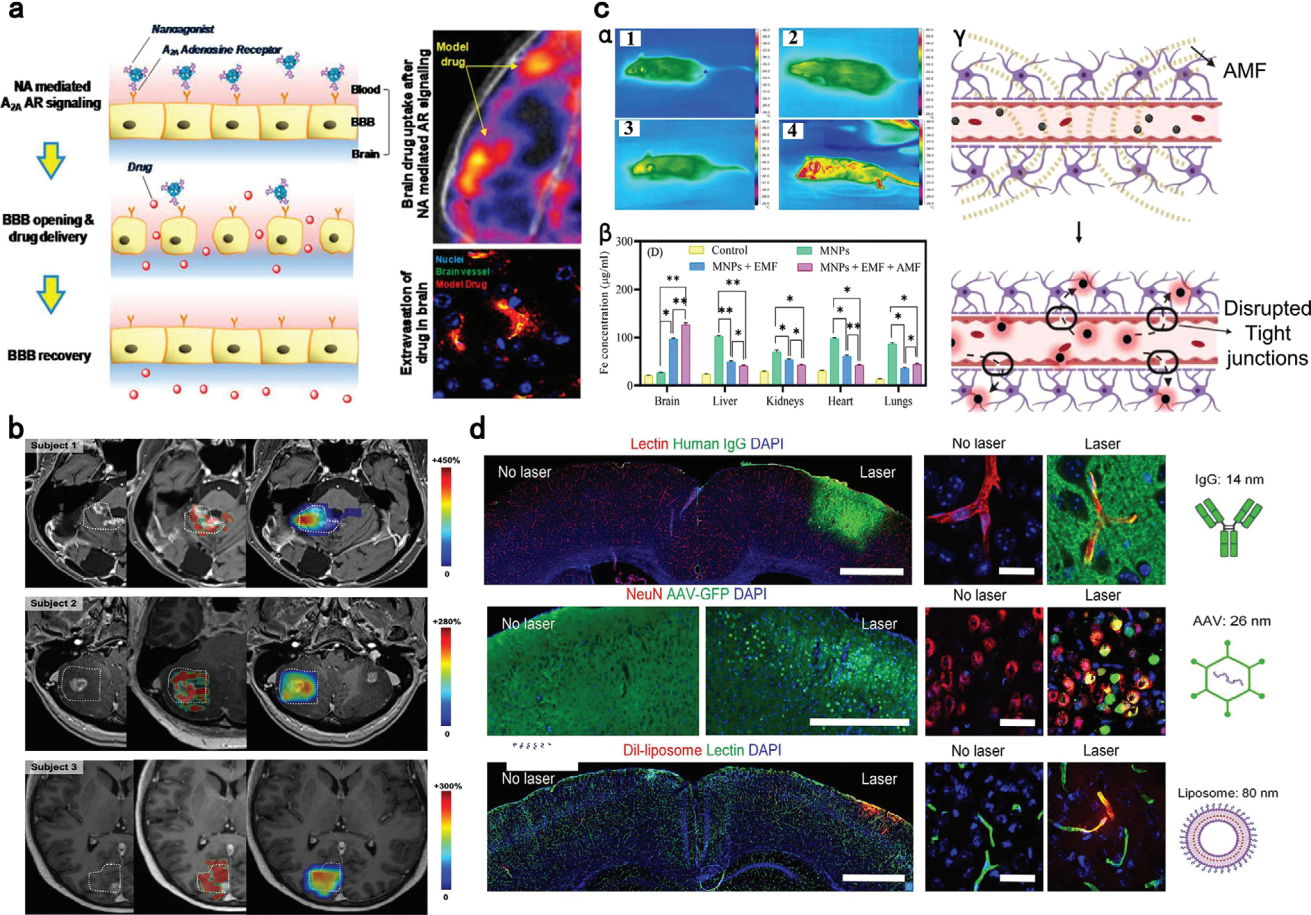
Biochemical and physical strategies to overcome BBB by enhancing paracellular transport. a) Scheme of nanoagonists‐mediated A_2A_ AR signaling to temporarily open TJs for enhancing brain delivery. Reproduced with permission.^[^ ^158^ ^]^ Copyright 2014, ACS publication. b) The effect of MRgFUS on ^111^In radiolabeled trastuzumab standardized uptake value ratio (SUVR) as measured by contrast‐enhanced T1‐weighted MRIs in three subjects. Left column: sonication volume (dotted white polygon); Middle column: resulting acoustic dose (color map); Right column: percentage difference in SUVR on delayed SPECT imaging (color map). The color bar displays the range of SUVR differences for each patient. Reproduced with permission.^[^ ^170^ ^]^ Copyright 2021, Meng et al., open access. c) Magnetic field exposure enhances the brain delivery of MNPs. (α) Thermal images of four C57BL/6 mice: (1) PBS; (2) MNPs; (3) MNPs + EMF; and (4) MNPs + EMF + AMF; (β) Determination of Fe in murine tissues by ICP‐MS. One‐way ANOVA analysis was adopted, ^*^ *p* < 0.1, ^**^ *p* < 0.01; (γ) Scheme of employing AMF to enhance BBB permeability of MNPs. Reproduced with permission.^[^ ^173^ ^]^ Copyright 2022, the Royal Society of Chemistry (RSC) publication. d) BBB modulation by laser pulse‐responsive AuNP‐BV11 enables the brain delivery of antibody, gene therapy vector, and liposome. Reproduced with permission.^[^ ^177^ ^]^ Copyright 2021, ACS publication.

Employing biochemical modulators to reversibly weaken the tightness of TJs/AJs also facilitates the BBB crossing.^[^ ^169^, ^170^ ^]^ The underlying mechanisms include direct alteration of TJs/AJs protein components (e.g., selective peptides and antibodies), modulation of the signaling pathways affecting TJs/AJs functions (e.g., Zot and adenosine receptor (AR) agonists), and interactions with their supporting environment or lipid rafts/membranes (e.g., calcium chelators, polycations, and cyclodextrins).^[^ ^169^, ^170^ ^]^ Notably, many of these modulators suffer from issues such as instability, rapid metabolism/clearance, low tissue selectivity and prolonged BBB compromise, leading to potential neurotoxicity.^[^ ^161^, ^170^, ^171^ ^]^ Integrating TJ modulators with nanoplatforms holds promise in enhancing their efficacy, tunability, safety and lesion‐targeting capability. For instance, Gao et al. developed a series of PEGylated dendrimer‐based nanoagonists (NAs) labeled with different numbers of Regadenoson (Reg) to open TJs and enhance BBB permeability (**Figure** **4**a).^[^ ^169^ ^]^ Reg is an FDA‐approved coronary vasodilator with potent and rapidly‐terminated activation on A_2A_ AR, making it a good candidate as a safe and effective TJ modulator. PEGylated dextran labeled with ^99^mTc‐DTPA chelator and rhodamine fluorophore were selected as the model drug, which has a molecular weight of 45 kDa and a hydrodynamic diameter of 7.4 nm. In vitro and in vivo studies attested that 30 min pretreatment of Den‐Reg16 greatly improved the blood‐to‐brain delivery of the model drug compared to free Reg. This enhancement was attributed to Reg's activation of A_2_A adenosine receptors, which reshapes endothelial cell cytoskeleton and downregulates TJ protein ZO‐1. Varying the number of Reg labels on NAs allowed for adjustable BBB opening durations, ranging from 0.5 to 2.0 h. Importantly, NAs demonstrated a satisfactory safety profile, avoiding issues like edema, apoptosis, and neuronal injuries observed with osmotic agents‐induced BBB penetration.^[^ ^172^ ^]^

To achieve controlled BBB crossing for safe brain delivery, external physical stimuli that enable noninvasive, temporary and spatially precise BBB penetration are highly desirable. MRgFUS represents one of the most promising physical stimuli to facilitate BBB opening.^[^ ^165^, ^173^, ^174^ ^]^ MRgFUS, in conjunction with ultrasound contrast agents like Definity and clinically available low‐power FUS devices, offers a non‐invasive, spatially precise and fine‐tuned method for BBB opening with no need to re‐engineer therapeutics.^[^ ^165^ ^]^ Low‐intensity sonification induces stable cavitation of contrast agents, making them expand and contract in sync with acoustic pressure. This process generates radiation force, mechanical stretching and push and pull interactions that enhance BBB permeability by compromising TJ structure and related signaling pathways. Additionally, MRgRUS enhances receptor‐, vesicle‐ or vacuole‐mediated transcytosis and the formation of fenestrations or channels in the BBB.^[^ ^175^, ^176^, ^177^, ^178^ ^]^ Several initial clinical trials have demonstrated the feasibility and efficacy of MRgFUS‐enhanced noninvasive, reversible and spatially‐targeted BBB opening in patients with amyotrophic lateral sclerosis (NCT03321487), Alzheimer's disease (AD, NCT03671889), Parkinson's disease (NCT03608553) and Her2‐positive brain metastases of breast cancer (NCT03714243).^[^ ^179^, ^180^, ^181^, ^182^ ^]^ Notably, MRgFUS enhanced trastuzumab uptake in 87 ± 17% of sonicated voxels by an average of 101% ± 71%, compared with only 8% ± 8% of voxels with −18% ± 26% in control lesions (Figure 4b). This resulted in a unidimensional tumor volume reduction by 19% ± 12%.^[^ ^181^ ^]^ Although no clinically significant adverse events were detected in these clinical trials, MRgFUS with high transducer frequency and ultrasound intensity can potentially cause varying degrees of hemorrhage, ischemia, inflammation and cerebral edema.^[^ ^165^ ^]^ Currently, a relatively safe FUS parameter for non‐human primates is within the range of 0.185–0.266 MPa with a 690 kHz transducer. In‐depth research is necessary to strike a balance between the safety and efficacy of MRgFUS‐induced BBB penetration for treating CNS diseases.^[^ ^165^, ^178^ ^]^

Another physical stimulus for promoting BBB penetration is EMF, especially when used in combination with magnetic nanoparticles (MNPs).^[^ ^183^, ^184^ ^]^ For example, Gupta et al. combined external magnetic fields and alternating magnetic fields (AMF) to enhance the brain delivery of MNPs. The external magnetic field initially guides MNPs to the targeted brain site from the blood stream, while AMF induced localized tissue heating, temporarily opening the BBB for enhanced brain delivery. This dual magnetic guidance resulted in the highest accumulation of MNPs in the brain, surpassing that in the liver, kidney and lung. In vitro studies revealed that the expression of TJ protein, ZO‐1, significantly downregulated after 1 h of AMF exposure and gradually returned to control levels after 2 and 3 h of AMF treatment, respectively (Figure 4c).^[^ ^184^ ^]^ Moreover, EMF facilitates BBB permeability by promoting transcytosis and downregulating efflux pump expression.^[^ ^185^, ^186^ ^]^ Nevertheless, EMF‐enhanced BBB penetration is still in its early stages, and further research is required to identify the optimal parameters of magnetic fields and nanomaterial properties to achieve efficient BBB traversal without safety concerns.^[^ ^161^, ^187^ ^]^

Photo stimulation can improve brain delivery in a spatiotemporally‐resolved and noninvasive manner, particularly when integrated with specific nanomaterials. The mechanisms underlying this approach involve the photoacoustic heating effect and mechanical wave generation induced by laser‐matter interactions.^[^ ^188^ ^]^ For instance, Li et al. developed a laser pulse‐responsive BV11‐targeting gold NPs (AuNP‐BV11) to reversibly modulate BBB permeability for enhanced brain‐targeted delivery. BV11 is a component of the TJ complex, and anti‐BV11 antibodies were attached to AuNPs (50 nm) to target TJ. Following transcranial picosecond laser excitation, AuNP‐BV11 can reversibly widen TJ to enhance BBB permeability. Pretreatment with AuNP‐BV11 and concurrent laser excitation significantly improved the local brain delivery efficiency of human IgG antibody, virus vector (AAV‐CamKII‐GFP) and liposome with sizes of 14, 26, and 80 nm, respectively (Figure 4d). Moreover, this approach exhibited no interference with spontaneous vasomotion, cerebral vasculature and brain parenchyma.^[^ ^188^ ^]^ In addition to laser excitation, NIR irradiation of NIR‐responsive (nano)materials has also been reported to generate hyperthermia for enhanced BBB penetration.^[^ ^189^ ^]^

**Figure 5:**
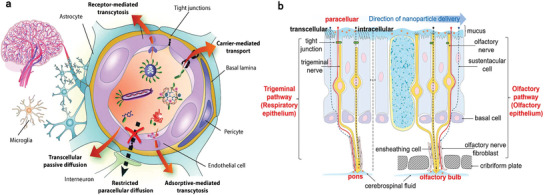
a) Various ligands can mediate transcytosis pathways for BBB penetration. Reproduced with permission.^[^ ^152^ ^]^ Copyright 2016, RSC publication. b) Nose‐to‐brain delivery to bypass BBB and enter the CNS system. Reproduced with permission.^[^ ^187^ ^]^ Copyright 2022, Elsevier publication.

The aforementioned strategies for crossing the BBB primarily rely on the paracellular pathway, in which widening TJ/AJ may provide access to hazardous substances, potentially leading to neurotoxicity. To circumvent these risks, various ligand‐triggered transcytosis pathways have been employed to enhance brain delivery efficiency (**Figure** **5**a).^[^ ^163^ ^]^ Cell‐penetrating peptides (CPPs), a group of peptides with amphipathic and/or cationic sequences, can electrostatically interact with cell membranes, mediating adsorptive‐mediated transcytosis.^[^ ^160^ ^]^ Representative CPPs include TAT, penetratin and oligoarginine, etc.^[^ ^163^, ^190^ ^]^ In contrast to CPPs, various ligands act on transporters and/or receptors to trigger transcellular pathways. An ideal targeted receptor/transporter for delivering cargoes to the brain parenchyma should possess the following features: 1) high expression on the luminal side of brain vasculature and low expression in other tissues; 2) mediation of transcytosis rather than confining cargoes inside endothelial cells; 3) high turnover from cell membranes; 4) broad substrate recognition and resistance to saturation by endogenous substrates.^[^ ^163^, ^173^ ^]^ Well‐studied ligands for receptor/transporter‐mediated BBB penetration comprise transferrin, OX26 antibody, THR peptide, and CRT peptide for transferrin receptor,^[^ ^163^, ^191^ ^]^ choline for choline transporters and ACh for ACh transporter,^[^ ^192^ ^]^ glucose for glucose transporters (GLUT1),^[^ ^164^, ^193^ ^]^ receptor‐associated protein (RAP12), angiopep‐2 or apolipoprotein E (ApoE) for lipoprotein receptor‐related protein‐1 (LRP1),^[^ ^163^, ^194^ ^]^ ApoB and ApoE for low‐density lipoproteins (LDLRs), glutathione (GSH) for GSH transporters,^[^ ^195^ ^]^ and rabies virus glycoprotein (RVG29) for nicotinic ACh receptors,^[^ ^196^ ^]^ etc. These ligands present varying BBB‐penetrating efficiency and selectivity when conjugated with different cargoes. Additionally, linear peptide‐based BBB shuttle ligands often face rapid degradation by proteases, a challenge that can be addressed through *retro‐enantio* approaches and chemical modifications.^[^ ^163^ ^]^

In addition to the strategies for BBB crossing, several BBB‐bypassing approaches have been developed. However, most of these methods involve invasive parenchymal or intraventricular injections directly into the surgery‐exposed brain or injections into the subarachnoid space.^[^ ^161^, ^197^ ^]^ These complicated and invasive procedures demand skilled surgeons or practitioners and carry a high risk of CNS injuries and poor patient compatibility. In contrast, intranasal delivery‐mediated BBB bypass is highly desirable due to its non‐invasiveness, rapid absorption, avoidance of hepatic first‐pass effects and patient compliance.^[^ ^161^ ^]^ Upon intranasal delivery, drugs initially penetrate the nasal mucosa and are transported along the trigeminal and olfactory nerves via intraneuronal and/or paracellular pathways, eventually reaching the pons and olfactory bulb. From there, they merge into the cerebrospinal fluid (CSF) circulation and gradually distribute to other regions of the brain parenchyma (Figure 5b).^[^ ^161^, ^198^ ^]^ Efficient nose‐to‐brain delivery requires addressing challenges such as rapid mucociliary clearance in the respiratory epithelium, enzymatic degradation in the nasal cavity and the unwanted entry of drugs into the nasopharynx and gastrointestinal tract.^[^ ^199^, ^200^ ^]^ Nanoplatforms with adjustable physicochemical properties such as size, morphology, composition and surface features, might offer viable solutions to these challenges.

#### 5.1.2 Postoperative Cognitive Dysfunction (POCD)

POCD is a prevalent neurological complication that arises following surgery and anesthesia. It is characterized by impairments in memory, learning and information processing capabilities and alterations in personality.^[^ ^33^ ^]^ The incidence rates of POCD are ≈25%–40% one week after surgery with anesthesia, decreasing to ≈10% three months post‐surgery.^[^ ^32^, ^33^ ^]^ Although POCD typically manifests as self‐limiting and subtle cognitive impairment, it can persist for years and potentially deteriorate into severe cognitive deficits. This is particularly concerning in elderly individuals with preexisting neurodegenerative diseases, as it is associated with high morbidity and mortality.^[^ ^32^, ^201^ ^]^ Unfortunately, therapeutic drugs for managing POCD have not been developed, partly due to the insufficient attention to POCD within the academic community and the elusive nature of its pathological mechanisms.^[^ ^32^, ^33^ ^]^

To date, several potential mechanisms underlying POCD have been identified, including neuroinflammation, aggregation of Aβ peptides and phosphorylated tau protein, mitochondrial dysfunction, oxidative stress, damage to synapses, and a shortage of neurotransmitter and neurotrophic support, etc.^[^ ^33^, ^202^, ^203^, ^204^ ^]^ Remarkably, POCD shares clinical symptoms with early‐stage AD, which has pivotal pathogenic hallmarks of Aβ plaques and p‐tau tangles.^[^ ^33^ ^]^ Despite the nebulous relationship between POCD and AD, efforts to develop treatments for AD may provide insights into therapeutics for POCD.

**Figure 6:**
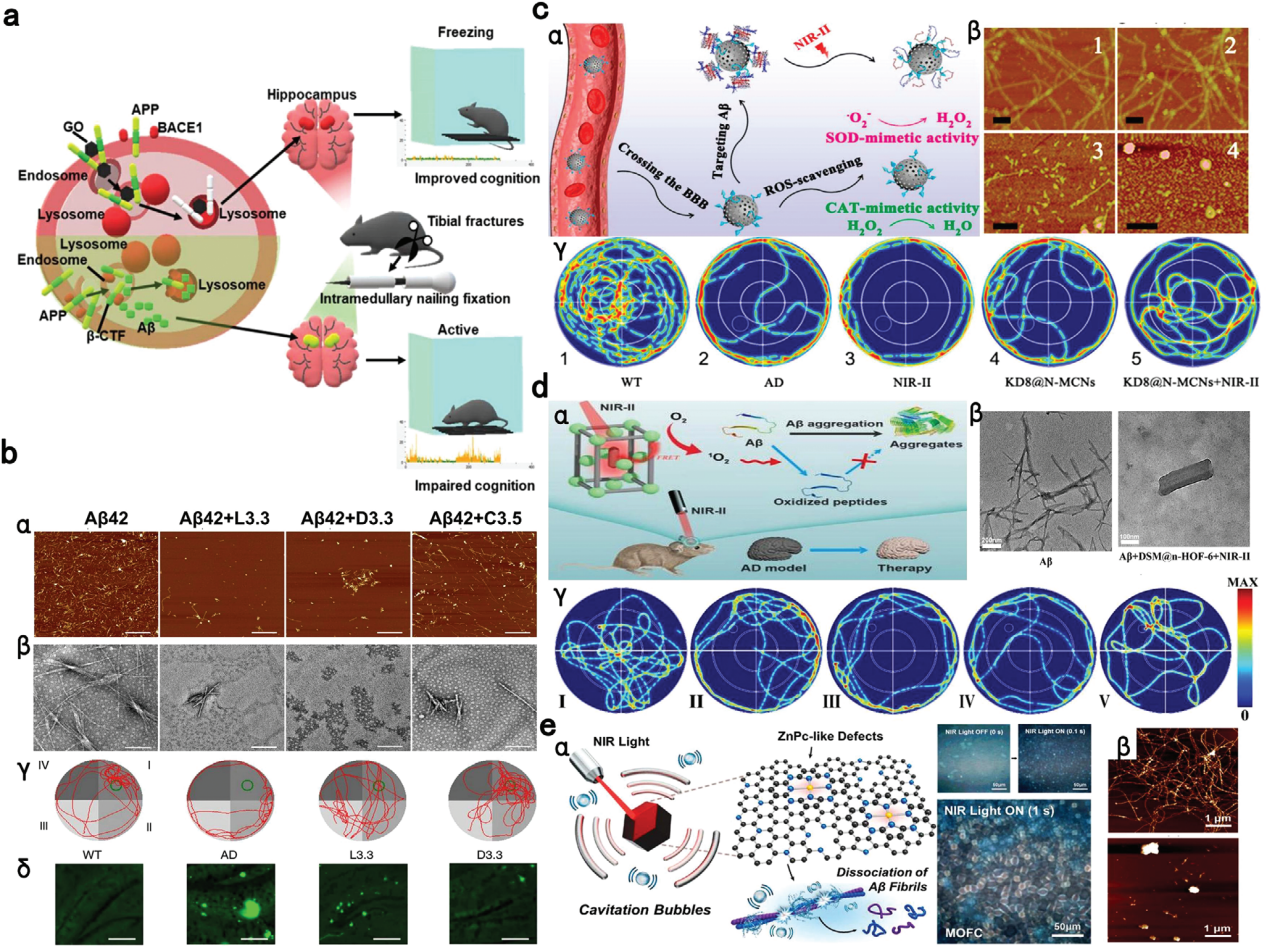
Modulating the proteostasis of Aβ protein can mitigate cognitive deficits. a) Schematic illustration of GO alleviating Aβ burden and improving POCD in mice. Reproduced with permission.^[^ ^146^ ^]^ Copyright 2020, Zhang et al., open access. b) Chiral GSH‐stabilized Au NPs rescue memory deficits in a mouse model of AD. AFM (α) and TEM (β) images of Aβ42 in the absence and presence of L3.3, D3.3, or C3.5 after co‐incubation for 48 h. Scale bars: 1 µm and 200 nm, respectively; (γ) Track of probe test; (δ) Immunofluorescence for Aβ in the hippocampus of different groups. Scale bars: 200 µm. Reproduced with permission.^[^ ^200^ ^]^ Copyright 2020, Hou et al., open access. c) Schematic illustration (α) of NIR‐II nanozyme KD8@N‐MCNs attenuating Aβ deposition and AD progression; (β) AFM images Aβ fibrils in different groups, 1: Aβ fibrils; 2: Aβ fibrils + NIR‐II; 3: Aβ fibrils + KD8@N‐MCNs; 4: Aβ fibrils + KD8@N‐MCNs + NIR‐II. Scale bars: 300 nm; (γ) Representative swimming paths of mice in the probe test. Reproduced with permission.^[^ ^203^ ^]^ Copyright 2020, ACS publication. d) Illustration (α) of NIR‐II‐excited catalyst inhibiting Aβ aggregation by photooxygenation; (β) TEM images of Aβ42, and Aβ42+DSM@n‐HOF‐6+NIR‐II; (γ) Representative swimming paths of mice in different groups. I: wild type; II: AD; III: AD+NIR‐II; IV: DSM@n‐HOF‐6; V: DSM@n‐HOF‐6+NIR‐II. Reproduced with permission.^[^ ^205^ ^]^ Copyright 2021, Wiley publication. e) Scheme (α) of photoacoustic dissociation of Aβ aggregate structure by NIR‐irradiated MOFC and video snapshots of the formation of cavitation bubbles; (β) AFM images of Aβ fibrils alone (top) and Aβ fibrils + NIR‐II light + MOFC (bottom). Note that the white spots in the AFM images represent the MOFC nanoparticle aggregates. Reproduced with permission.^[^ ^202^ ^]^ Copyright 2022, ACS publication.

Owing to their unique nano‐bio interactions, inherent bioactivities, large surface area, drug loading capacity, adjustable physicochemical properties, and enhanced BBB penetration, versatile nanomedicines have demonstrated therapeutic potential in mitigating cognitive impairments, including POCD, AD and dementia. Nanomedicines have been reported to attenuate POCD and AD by modulating the production, folding, aggregation and degradation of pathogenic Aβ.^[^ ^205^, ^206^, ^207^ ^]^ For instance, owing to the higher affinity of graphene oxide nanosheets (GO) for Aβ precursor protein (APP) than for Aβ‐activating enzymes (BACE1), GO interfered with the co‐localization of APP and BACE1 and inhibited the β‐cleavage of APP by BACE1, thus decreasing Aβ production. Moreover, GO treatment improved the transport of endosomal cargo to lysosomes, enhancing Aβ degradation (**Figure** **6**a). Bilateral intracerebral injection of GO decreased cognitive dysfunction of POCD not only by inhibiting Aβ production (which can be decreased by BSA coating, BGQ) but also by increasing Aβ lysosomal degradation (which can be blocked by lysosomal degradation suppressor, CQ).^[^ ^157^ ^]^ Additionally, molecular dynamics simulations and ex vivo experiments have indicated that pristine graphene and GO nanosheets can suppress the fibrillation of Aβ monomers and clear mature Aβ fibrils.^[^ ^208^ ^]^ Notably, pristine nanomaterials will form a dynamic bio‐corona on their surfaces in biological environments, which can either enhance or inhibit their anti‐Aβ performance.^[^ ^205^, ^206^ ^]^ Furthermore, the size, morphology, core composition, and surface properties (e.g., hydrophobicity, charge, roughness, surface coatings, bio‐corona) of nanomaterials collectively influence their interaction with Aβ and, consequently, their therapeutic efficacy.^[^ ^205^ ^]^ To enhance the therapeutic efficiency against cognitive impairment, modifying small‐size nanomaterials with multifunctional biomolecules is a promising strategy.^[^ ^207^, ^209^ ^]^ For instance, Hou et al fabricated L‐GSH and D‐GSH stabilized Au NPs with different sizes for AD treatment.^[^ ^209^ ^]^ GSH‐decorated Au NPs were prepared by the reduction of HAuCl_4_ with NaBH_4_ in the presence of fresh L‐GSH or D‐GSH. Surface functionalization with GSH imparts several advantages to Au NPs: 1) stability for dispersion in the systemic circulation; 2) enhanced BBB penetration through receptor‐mediated transcytosis; and 3) high affinity for Aβ monomers. D‐GSH‐modified Au NPs with a size of 3.3 nm (D3.3) exhibited the strongest capability to capture Aβ monomers and prohibit Aβ aggregation. Intravenous administration of D3.3 and L3.3 presented 10 times higher brain accumulation than those in the control group, with sustained presence in the brain for over 48 h. A four‐week intravenous injection of D3.3 greatly declined Aβ aggregation and rescued memory impairment in an AD model (Figure 6b). Besides linear peptides, Aβ‐targeting cyclic peptides with better chemical, thermal, and proteolytic stability have been developed based on the nucleating sequence of Aβ or other strategies, such as cyclo‐[KLVFF], cyclic LK7, etc. Further details regarding the design and synthesis of cyclic peptides and their interactions with amyloidogenic proteins can be found in this reference.^[^ ^207^ ^]^

Furthermore, nanomaterials with unique optical and electronic properties have demonstrated the ability to inhibit Aβ aggregation and promote fibril dissociation by eliciting photothermal, photodynamic and photoacoustic effects.^[^ ^210^, ^211^, ^212^, ^213^ ^]^ However, these NIR‐mediated phototherapies are double‐edged swords, as off‐target photothermal and photooxidative effects may potentially harm normal tissues. Consequently, there is a growing interest in the development of second NIR (1000–1700 nm)‐excited photosensitizers with Aβ‐targeting ability, offering lower phototoxicity, enhanced deep tissue penetration, and precise targeting capabilities. For instance, Ma et al. reported a KLVFFAED (KD8)‐grafted N‐doped mesoporous carbon nanosphere (KD8@N‐MCNs), which was fabricated following three sequential steps, including preparation of N‐MCNs by a pyrolysis approach, carboxyl modification of N‐MCNs, and KD8 covalent graft. KD8@N‐MCNs attenuated AD severity by efficiently crossing BBB, targeting Aβ deposits, scavenging ROS and converting NIR‐II (1064 nm) radiation into heat for decreasing Aβ aggregation (Figure 6c).^[^ ^212^ ^]^ Similarly, Zhang et al. encapsulated pyridinium hemicyanine dye (DSM) into porphyrin‐based hydrogen‐bonded organic framework (HOF) by a one‐pot approach to yield a NIR‐II photooxygenation catalyst (DSM@n‐HOF‐6) for the attenuation of AD.^[^ ^214^ ^]^ KD8 peptides were further conjugated onto the surface of DSM@n‐HOF‐6 to facilitate BBB penetration and Aβ binding. Intraperitoneal injection of DSM@n‐HOF‐6@KD8, coupled with NIR‐II radiation (1040 nm), led to enhanced and prolonged brain accumulation, weak photothermal effects, robust photooxidative effects, diminished Aβ deposits and improved cognitive functions (Figure 6d).^[^ ^214^ ^]^ More recently, Jang et al. fabricated a metal‐organic framework‐derived carbon (MOFC) by carbonizing crystalline zeolitic imidazolate framework‐8 polyhedrons in an N2 atmosphere at 800 °C with NaOH etching. The resulting MOFC exhibited NIR‐excited photoacoustic capability for disassociating Aβ aggregates.^[^ ^211^ ^]^ Under NIR irradiation, MOFC absorbs and converts photon energy into acoustic waves, promoting the vaporization of surrounding liquid water molecules to produce cavitation bubbles. The resulting strong hydrodynamic stress, due to bubble collapse induces asymmetric distribution of water molecules, thus facilitating the dissociation of Aβ aggregates (Figure 6e).^[^ ^211^ ^]^ Despite commendable in vitro outcomes, further in vivo experiments are warranted to assess the efficacy and safety of these anti‐Aβ phototherapies.

**Figure 7:**
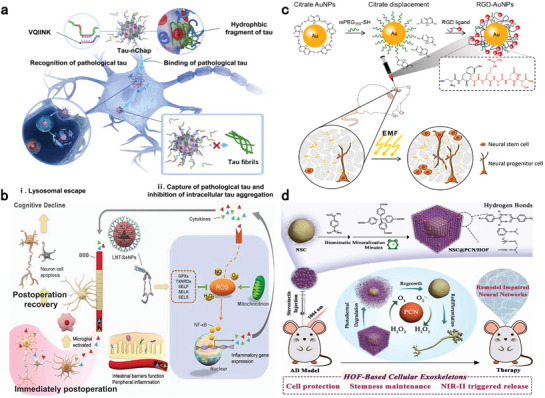
Novel strategies for the alleviation of cognitive impairments. a) Scheme of Tau‐nChap selectively capturing intracellular pathological tau and inhibiting their aggregation in neurons. Reproduced with permission.^[^ ^214^ ^]^ Copyright 2022, Elsevier publication. b) Scheme of the pathophysiological features of POCD and potential effects of LNT‐SeNPs. Reproduced with permission.^[^ ^144^ ^]^ Copyright 2022, Elsevier publication. c) Schematic illustration of in vivo neurogenesis induced by intracerebrally injected electromagnetized AuNPs stimulated by the EMF. Reproduced with permission.^[^ ^225^ ^]^ Copyright 2021, Elsevier publication. d) Scheme of the formation and removal of HIF shells on individual NSCs and the mechanism of remodeling impaired neural networks for AD treatment. Reproduced with permission.^[^ ^228^ ^]^ Copyright 2022, Wiley publication.

The disappointing clinical outcomes of various anti‐Aβ therapeutics have shifted researchers’ interests toward tau‐based therapy for treating cognitive deficits.^[^ ^215^ ^]^ Tau protein plays a pivotal role in maintaining microtubule stabilization during neuronal transport. Tau phosphorylated at threonine 217 (tau‐PT217) and 181 (tau‐PT181) have been identified as plasma biomarkers for the early diagnosis of AD and postoperative delirium.^[^ ^216^ ^]^ Moreover, anesthesia and surgery have been shown to acutely increase the release of tau‐PT217 from pulmonary plasmocytes to the blood stream, which is further transported to the brain and increases neuronal excitability to cause postoperative delirium.^[^ ^216^ ^]^ Sevoflurane was revealed to induce cognitive deficits by enhancing tau/p‐tau levels in various bioenvironments and facilitating the trafficking of tau/p‐tau from extracellular vesicles to microglia to trigger neuroinflammation.^[^ ^204^ ^]^ As cognitive decline progresses, hyperphosphorylated and aggregated tau proteins form intracellular neurofibrillary tangles (NFTs), which are closely associated with neuronal death, synaptic dysfunction and the severity of cognitive disorder, surpassing the relevance of extracellular Aβ plaques.^[^ ^217^, ^218^, ^219^ ^]^ To date, several nanomedicines have been developed to inhibit tau phosphorylation and aggregation, while facilitating the degradation of NFTs.^[^ ^215^, ^220^, ^221^, ^222^, ^223^ ^]^ For instance, Xu et al. designed a biomimetic tau‐nanochaperone (Tau‐nChap) with tau‐targeting ability for AD treatment (**Figure** **7**a).^[^ ^223^ ^]^ VQIINK, derived from the tau sequence, is a strong driving force for tau aggregation and NFT formation, showing selective binding to pathological tau. To form Tau‐nChap, VQIINK was first covalently conjugated onto the amphiphilic copolymer PEG‐block‐poly(ε‐caprolactone) to form VQIINK‐PEG‐b‐PCL, which co‐assembled with pH‐responsive poly (βamino ester)‐block‐PCL (PAE‐b‐PCL) in weak acidic solution, thereby generating micelles with hydrophobic cores composed of PCL and mixed shells consisting of VQIINK‐PEG and PAE. After adjusting the pH 7.4, the outstretched PAE chains deprotonated to form hydrophobic microdomains around the micellar surface, which further enhances tau capture through hydrophobic interactions. Moreover, Tau‐nChap possesses efficient lysosomal escape capabilities, allowing it to reach the cytoplasm for effective inhibition of tau aggregation. In comparison, MSPM represents the polymeric micelle without VQIINK decoration, while Tau‐PM is a nanochaperone devoid of PAE. Among the three nanoplatforms, Tau‐nChap exhibited the highest dissociating ability on tau tangles after incubation at 37 °C for 7 days. Novel object recognition (NOR) tasks and Morris water maze (MWM) tests suggested that intracerebral injection of Tau‐nChap significantly mitigated the spatial learning and memory impairments in AD mice. Other Tau‐targeting motifs include clinically available T807 (AV‐1451),^[^ ^221^ ^]^ non‐natural D‐peptide TLK,^[^ ^215^ ^]^ DMK6240,^[^ ^224^ ^]^ and anti‐tau antibodies,^[^ ^225^ ^]^ all of which selectively bind hyperphosphorylated tau aggregates without interfering with normal tau's physiological functions. Moreover, tau aggregating inhibitors (e.g., methylene blue), ROS scavengers (e.g., curcumin, polyphenols, catechins, quercetin and antioxidative nanozymes) and autophagy inducers (e.g., nanoceria) have been integrated with nanoplatforms to enhance the therapeutic efficacy of anti‐tau treatments.^[^ ^217^, ^221^, ^222^, ^225^ ^]^

Single‐target therapies based on anti‐amyloid or anti‐tau mechanisms may encounter limitations in therapeutic efficacy since the presence of amyloid plaques and NFTs heralds moderate to severe cognitive degeneration. To address POCD or AD at an early stage, multi‐target therapies designed to synergistically modulate oxidative stress, neuroinflammation, proteostasis of Aβ and tau, neuron growth and regeneration, or the brain‐gut axis have demonstrated promising therapeutic potential.^[^ ^155^, ^226^, ^227^, ^228^ ^]^ Nanomedicines have emerged as versatile platforms capable of achieving one or multiple of these functions, primarily relying on the bioactivities and nano‐bio interactions derived from nanomaterials’ inherent properties and/or encapsulated drugs.^[^ ^154^, ^157^, ^223^, ^227^, ^228^, ^229^, ^230^ ^]^ Versatile nanozymes, exhibiting one or multiple enzyme‐mimetic activities such as peroxidase (POD), catalase (CAT), Superoxide dismutase (SOD), glutathione peroxidase (GPx) or glucose oxidase (GOx) have been reported to mitigate CNS diseases due to their inherent antioxidation and anti‐inflammation activity. These nanozymes encompass diverse categories, including metal‐based (e.g., Au NPs, AuAg alloy NPs), metal oxide‐based (e.g., CeO_2_ NPs, Mn_3_O_4_ NPs), carbogenic (e.g., carbon nanotubes, graphene oxide) and single‐atom nanozymes (e.g., Pt/CeO_2_). Nanozymes’ catalytic capabilities primarily arise from factors such as a high degree of exposed active surface atoms, nanoscale sizes, multivalence state transformations and unique electronic structures.^[^ ^154^, ^231^ ^]^ For example, Cai et al. constructed a novel hollow manganese Prussian white nanocapsule (HMPWCs) nanozymes for attenuating AD. Due to the low redox potential and multivalent transformation of Mn^2+^ and [Fe(CN)6]^4−^, HMPWCs exhibited a robust ROS scavenging capability, effectively eliminating OH·, H_2_O_2_ and O_2_^−^·. It was revealed that the right hippocampal microinjection of HMPWCs in AD‐like rats significantly improved spatial learning and memory in these rats by synergistically relieving oxidative stress, reducing p‐Tau levels via the activation of AKT/GSK‐3β and deactivation of ERK, and inhibiting the activation of microglia and astrocytes and release of pro‐inflammatory cytokines.^[^ ^227^ ^]^ Oral administration may offer advantages over invasive injection regarding patient compatibility and gut‐brain regulation. Liang et al. reported a facile and large‐scale producible lentinan‐modified selenium NPs (LNT‐SeNPs) for efficient POCD treatment (Figure 7b).^[^ ^155^ ^]^ LNT‐SeNPs was fabricated by mixing Na_2_SeO_3_ and LNT in vitamin C solution. Oral administration of LNT‐SeNPs increased the expression of selenoprotein‐based reducing enzymes in the serum and hippocampus of aged mice. A thirty‐day daily oral supplementation regimen of LNT‐SeNPs, initiated prior to laparotomy operation and continued until the mice were sacrificed, resulted in significant POCD alleviation with less anxiety‐like behavior and improved recognition memory and spatial learning and memory, as indicated by the open field test, NOR test and Barnes maze test. Additionally, LNT‐SeNPs + surgery group exhibited inhibition in caspase8‐dependent neuronal apoptosis, surgical‐induced oxidative stress, proinflammatory cytokine release, microglial activation, and intestinal microbiota disturbance. Similarly, another selenium‐based nanomedicine, TGN‐CGA@SeNCs with brain‐targeting peptide (TGN) anchoring and chlorogenic acid loading (CGA, a neuroprotective polyphenol with low bioavailability) decelerated AD progression. This was achieved through enhanced brain distribution of TGN‐CGA@SeNCs following oral administration, inhibition of Aβ aggregation and neuroinflammation, and regulation of brain glucose homeostasis and gut microbiota.^[^ ^228^ ^]^

In addition to addressing the aforementioned pathogenetic factors, promoting neuron growth and regeneration is highly desirable for cognitive improvement. Nanomedicines, either through their inherent bioactivity or by delivering neuroprotective peptides, genes, or stem cells, have demonstrated potential in promoting neurogenesis to treat cognitive deficits. For instance, tetrahedral framework nucleic acid has been reported to treat trauma‐induced cognitive impairment by promoting the proliferation of endogenous neural stem cells (NSCs) while inhibiting neuroinflammation.^[^ ^232^ ^]^ Xiao et al. conjugated graphene quantum dots (GQDs) with neuroprotective peptides to form GQDG, which decreased Aβ plaques, inhibited neuroinflammation, protected synapse structure and promoted neurogenesis to mitigate AD severity.^[^ ^226^ ^]^ Furthermore, nanomedicines can augment the therapeutic efficacy of NSCs against cognitive deficits. For example, alanine‐bearing fullerene has been reported to enhance the growth and differentiation of NSCs into neurons and protect NSCs against oxidative damage by improving NSC's antioxidant systems, including SOD, GSH‐Px and GSH.^[^ ^233^ ^]^ Additionally, physical stimulus‐responsive nanodrugs provide opportunities to regulate the proliferation and differentiation of NSCs. In the existence of EMF, intracerebral injection of magnetizable RGD‐conjugated Au NPs has been shown to increase hippocampal neurogenesis in both adult normal mice, aged mice and Hutchinson–Gilford progeria mice models. These effects are mediated by activating Kat2a, a histone acetyltransferase, to promote epigenetic modifications, cellular plasticity and cognitive improvements (Figure 7c).^[^ ^234^ ^]^ Besides EMF, light stimulation is another widely used approach to modulate NSCs, including genetic and nongenetic optical modulation.^[^ ^235^ ^]^ Reportedly, NSCs constitutionally express blue‐ and red‐sensitive photoreceptors (opsins) that can trigger NSCs’ growth and differentiation, respectively. Intracerebral injection of upconversion NPs, which convert deep‐penetrating NIR light to blue light, has been reported to noninvasively regulate in vivo NSCs differentiation.^[^ ^235^ ^]^

Nanomaterials have emerged as facilitators for NSC transplantation in the treatment of cognitive dysfunctions by guiding NSC differentiation and providing a protective microenvironment. For example, co‐delivery of small interfering RNA (SiSOX9), retinoic acid (RA) and Ce NPs through a H_2_O_2_‐responsive MOF, termed ‘CeRMS’ were used for transfecting NSCs, which were subjected to stereotactic injection for AD treatment. The knockdown of SOX9 inhibited gliogenesis, while RA promoted neurogenesis, and nanoceria alleviated oxidative stress to protect NSCs. Consequently, this approach leads to improved neurogenesis and increased survival of newborn neurons, effectively mitigating cognitive dysfunctions in AD mice.^[^ ^236^ ^]^ More recently, Yu et al. reported a thermal‐responsive RA‐loaded porous carbon nanospheres (RA@PCNs)‐doped HOF nanocarriers (denoted ‘NSC@PCN/RA/HOF’) based on a biomineralization method for improved transplantation of NSCs to treat AD.^[^ ^237^ ^]^ SOD‐ and CAT‐mimetic PCNs with NIR‐II photothermal conversion ability were mineralized with HOF to create artificial cellular exoskeletons, which can protect encapsulated NSCs from membrane damage and oxidative stress, maintain NSCs stemness and enable on‐demand NSCs release upon NIR irradiation. Furthermore, RA upregulates the expression of neurogenesis‐associated genes in NSCs. With NIR irradiation, bilateral stereotactic transplantation of NSC@PCN/RA/HOF into the hippocampus of AD mice significantly ameliorates cognitive impairments, promoting neurogenesis and neuroprotection (Figure 7d).^[^ ^237^ ^]^

#### 5.1.3 Opioid‐Related Complications

Nanomedicines have been developed to address various complications associated with opioid use, including acute and chronic toxicity like respiratory depression or apnoea,^[^ ^238^ ^]^ constipation,^[^ ^239^ ^]^ opioid tolerance, hyperalgesia and addiction.^[^ ^21^, ^240^, ^241^, ^242^ ^]^ In this context, nanomaterials serve as drug delivery systems to improve the solubility, stability, and bioavailability of therapeutic molecules and refine their PK profiles.

**Figure 8:**
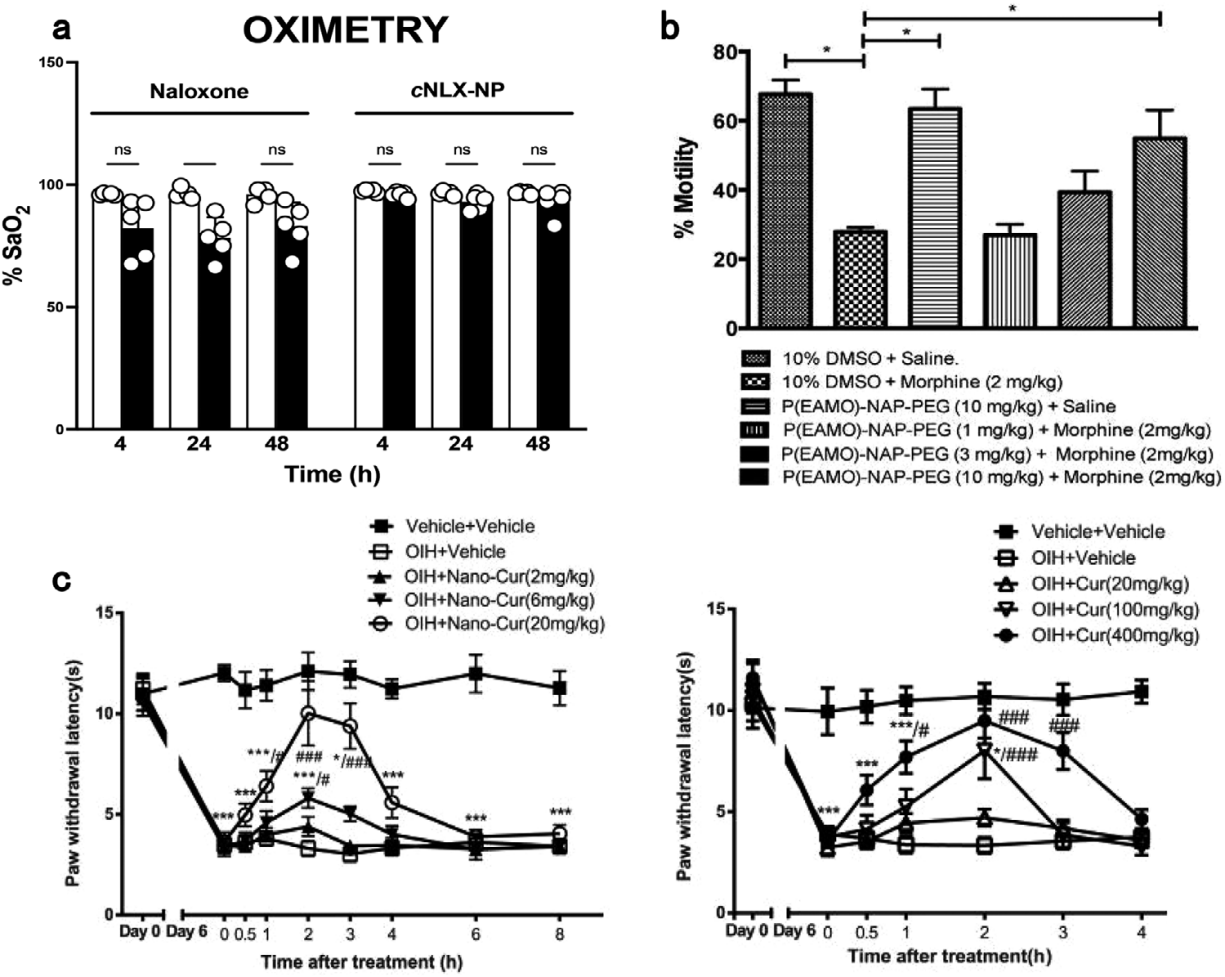
Versatile nanomedicines decreased various opioid‐induced complications. a) cNLX‐NP protected against fentanyl‐induced respiratory depression as indicated by Oximetry. Blank bar: Baseline; Black bar: post‐fentanyl, data are expressed as mean ± SD. Reproduced with permission.^[^ ^229^ ^]^ Copyright 2021, ACS publication. b) The effects of P(EAMO)‐NAP‐PEG on intestinal motility in acute morphine‐treated mice after oral administration. Data presents Mean ± SEM, n = 4–9, ^*^ *p* < 0.05. Reproduced with permission.^[^ ^230^ ^]^ Copyright 2017, ACS publication. c) Reversal of morphine‐induced OIH (thermal hyperalgesia) by PLGA‐curcumin (left) and non‐formulated curcumin (right). Data presents as Mean ± SEM. ^*^ *p* < 0.05; ^**^ *p* < 0.01, ^***^ *p* < 0.001 compared with vehicle + vehicle group; ^#^ *p* < 0.05, ^###^ *p* < 0.001 compared with the OIH + vehicle group. Reproduced with permission.^[^ ^232^ ^]^ Copyright 2016, Hu et al., open access.

Naloxone, a classical MOR antagonist, is the only clinically available therapeutic for alleviating acute opioid crisis. However, its therapeutic potential is severely restricted due to several drawbacks. After injection, naloxone undergoes rapid metabolism and inactivation, resulting in a short half‐life time, much shorter than that of potent synthetic opioids, for example, fentanyl. Consequently, opioid toxicity often reoccurs, necessitating repeated naloxone administration, which can lead to precipitated opioid withdrawal (POW) symptoms.^[^ ^151^, ^238^, ^243^ ^]^ To address this issue, developing a long‐acting naloxone formulation can avoid repeated administration, reducing the risks of opioid toxicity relapse and withdrawal. By learning lessons from Vivitrol, a marketed extended‐release noncovalent nano‐formulation of naltrexone, covalently loaded naloxone NPs were believed to prevent drug burst release and improve drug loading capacity and stability.^[^ ^238^, ^243^ ^]^ For example, naloxone‐poly(L‐lactic acid)‐based nanoplatform (Nal‐cNPs) exhibited extended reversal of high dose morphine‐induced analgesia in a mouse model of neuropathic injury for up to 98 h, four times longer than free naloxone.^[^ ^244^ ^]^ Both low and high doses of Nal‐cNPs were found to mitigate POW behavior more effectively than free naloxone in morphine‐dependent mice, which can be attributed to the slow, linear release of naloxone from Nal‐cNPs.^[^ ^243^ ^]^ A similar long‐acting naloxone nano‐formulation was developed to address fentanyl overdose. This effort is important as fentanyl is widely used for perioperative pain management and can elicit severe toxicity under relatively low doses (LD50, 1–3 mg kg^−1^).^[^ ^128^ ^]^ Specifically, poly(lactic‐co‐glycolic acid) (PLGA)‐naloxone copolymer‐based NPs (cNLX‐NPs) exhibited good drug loading capacity, prolonged half‐life and protection against repeated fentanyl challenges.^[^ ^238^ ^]^ Compared to the rapid elimination of free naloxone, cNLX‐NPs demonstrated a 34‐fold increase in half‐time and a 21‐fold increase in mean residence time. A single intramuscular injection of cNLX‐NPs was as effective as free naloxone in reversing immediate fentanyl effects and provided more prolonged protection (at least 48 h) against repeated fentanyl‐induced respiratory suppression and antinociception (**Figure** **8**a).^[^ ^238^ ^]^ The preparation of Nal‐cNPs and cNLX‐NPs started with the synthesis of naloxone‐containing copolymers by a organocatalyzed ring opening polymerization, which was converted to NPs by nanoprecipitation‐solvent displacement technique.

Despite the availability of marketed drugs for opioid‐induced constipation, such as methylnaltrexone, avlimopan, and 6β‐naltrexol, these periphery‐selective MOR antagonists still require improvement regarding efficacy and safety due to their relatively low MOR selectivity.^[^ ^239^ ^]^ To this end, Xu et al. identified a potent peripherally selective MOR antagonist named NAP with 100‐fold higher specificity over the delta and kappa opioid receptors. Nanoconjugate of NAP improved the peripheral selectivity, stability and local concentration of NAP, leading to a significant mitigation of morphine‐induced constipation with an oral administration of 10 mg kg^−1^ (Figure 8b).^[^ ^239^ ^]^ However, the safety and clinical efficacy of nano‐NAP conjugate should be further evaluated.

Long‐term opioid administration for chronic pain management can induce opioid tolerance, requiring a 30% to 50% dose escalation to maintain the same level of analgesia.^[^ ^63^ ^]^ To combat morphine tolerance, dendrigraft poly‐l‐lysine (DGL)‐PEG/dermorphin polymeric NPs were developed to load siRNA based on the electrostatic interaction for modulating the brain uptake for morphine and its metabolites (M3G and M6G).^[^ ^245^ ^]^ Dermorphin, an opioid peptide recognizing MOR expressed in brain parenchyma, is chosen as the brain‐targeting modality for enhanced BBB penetration. DGL‐PEG/dermorphin/siRNA not only stabilizes siRNA in the bioenvironment but also greatly enhances siRNA's brain delivery efficiency. Moreover, encapsulated siRNA is used to knock down OATP2B1, a transporter located on BBB endothelial cells responsible for mediating the brain uptake of morphine and M6G. Intrathecal injection of DGL‐PEG/dermorphin/siRNA downregulated OATP2B1 expression, effectively blocking morphine and M6G transport into the brain. This effect may further restore the expression/modification of MOR and Ca^2+^/calmodulin‐dependent protein kinase II α (CMKIIα), leading to the reversal of acute morphine tolerance.^[^ ^245^ ^]^

Opioid‐induced hyperalgesia (OIH), a major adverse event associated with prolonged opioid use, exacerbates nociception severity as opioid dosage increases.^[^ ^246^ ^]^ Overexpressed CaMKIIα in the superficial dorsal horn of the spinal cord plays a pivotal role in OIH pathogenesis. Hence, OIH may be attenuated by genetic‐related CaMKIIα downregulation or chemical‐induced inactivation.^[^ ^241^ ^]^ In this regard, Hu et al. reported that intrathecal injection of curcumin protected against OIH via repressing the over‐activated CaMKIIα. PLGA‐curcumin was further prepared by a multi‐inlet vortex mixer method. Compared to gavage 100–400 mg kg^−1^ free curcumin to achieve significant OIH attenuation, orally administered 6–20 mg kg^−1^ PLGA‐curcumin resulted in a corresponding protection against OIH, attributed to improved solubility and bioavailability of curcumin after nano‐formulation (Figure 8c).^[^ ^241^ ^]^

Opioid restatement, dependence and addiction are global health concerns and social and economic burdens, contributing to an estimated rate of 8.6 deaths per 1000 person‐years.^[^ ^247^ ^]^ Current outpatient treatments for opioid addiction primarily involve the maintenance treatment of opioid agonists (e.g., methadone and buprenorphine), and oral administration of high‐affinity and long‐lasting opioid antagonist, naltrexone.^[^ ^247^, ^248^ ^]^ However, these treatments have limitations, including poor adherence and life‐long agonist dependence in the case of opioid agonist maintenance, and low patient compliance and high dropout rates for regular naltrexone‐based detoxification.^[^ ^247^ ^]^ To this end, nanotechnology‐enabled sustained‐release formulations of naltrexone have demonstrated favorable therapeutic efficacy and patient compliance. Notably, a naltrexone‐loaded polylactide nanosuspension has been approved for clinical use to treat opioid and alcohol dependence (i.e., Vivitrol). This sustained‐release formulation allows one‐month therapeutic antagonizing action after a single shot into the gluteus muscle.^[^ ^247^, ^249^ ^]^

Opioids induce addiction partially by enhancing presynaptic release of excitatory neurotransmitters (e.g., glutamate and dopamine) and reinforcing several downstream pathways. Therefore, suppressing the release of excitatory neurotransmitters and related downstream pathways holds promise in combating opioid dependence. Milanesi et al. fabricated ferulic acid (FA)‐loaded nanocapsules (FA‐Nc) by an interfacial deposition‐solvent evaporation method. FA‐Nc improved FA's therapeutic potential in inhibiting morphine‐induced oxidative stress, regulating dopaminergic neurotransmission and deactivating the chronic addiction cascade, leading to significant mitigation in morphine craving.^[^ ^250^ ^]^ The suppression of α‐amino‐3‐hydroxy‐5‐methyl‐4‐isoxazolepropionic acid receptor (AMPAR), which mediates the rapid propagation of excitatory signals, has shown promising results in alleviating morphine craving. Topiramate (TPM), an AMPAR antagonist, is widely used for treating epilepsy, bipolar disorder and psychostimulant addiction, despite its high risk of impairing cognitive functions. TPM‐encapsulated chitosan (CS) NPs were prepared by ionotropic gelation of TPM‐containing CS with tripolyphosphate and Pluronic F‐68. TPM‐CS was suggested to prevent morphine reinstatement, regulate dopamine and glutamate neurotransmission and avoid cognitive deficits.^[^ ^251^ ^]^ Additionally, adenosine 3’, 5’‐monophosphate‐regulated phosphoprotein (DARPP‐32) plays a crucial role in regulating downstream neurobiological alterations following the release of excitatory dopamine and glutamate. DARPP‐32 siRNA therapy has been developed to defeat drug addiction.^[^ ^242^ ^]^ For instance, Bonoiu et al. developed a DARPP‐32 siRNA‐loaded gold nanorod (GNR‐DARPP‐32 siRNA) to counteract drug addiction. Specifically, GNR‐DARPP‐32 siRNA was prepared by seed‐mediated growth of HauCl_4_ in cetyltrimethylammonium bromide solution, followed by multilayer growth of cationic polyelectrolytes to electrostatically absorb siRNA. The introduction of GNR improved the stability and BBB‐penetrating capability of siRNA. This nanocomplex effectively downregulated the expression of DARPP‐32 and its downstream effectors in dopaminergic neurons.^[^ ^242^ ^]^ Further animal research confirmed that intracerebral administration of sustained‐release GNR‐DARPP‐32 siRNA greatly suppressed the expression of DARPP‐32 and related downstream effectors, including protein phosphatase‐1 and transcriptional factor CREB while increased phosphorylated CREB level in the brain periaqueductal gray area and ventral tegmental area from day 5 to day 15 post‐injection. Consequently, GNR‐DARPP‐32 siRNA treatment protected morphine‐addicted mice against morphine withdrawal syndrome without inducing conditioned place aversive behaviors.^[^ ^252^ ^]^

#### 5.1.4 Epilepsy

Epilepsy is a prevalent and chronic neurological disorder characterized by hyperactive neurons and brain circuits with synchronous electrical discharges.^[^ ^159^, ^253^ ^]^ While the occurrence of seizures in the perioperative period is relatively low, it remains imperative to comprehend the antiepileptic or pro‐epileptic properties of anesthetics.^[^ ^159^ ^]^ Most anesthetics exert suppressive effects on neurological processes and possess varying degrees of anticonvulsant properties. For example, benzodiazepines, barbiturates, propofol, isoflurane, and desflurane have potent anticonvulsant activity and can be safely employed in patients with seizures. Conversely, regular doses of etomidate and ketamine, high doses or rapid administration of opioids, enflurane, sevoflurane, and local anesthetics have proconvulsant effects, posing a risk of inducing epilepsy and should be avoided for seizure patients.^[^ ^159^, ^254^ ^]^

In the perioperative management of epilepsy, a comprehensive approach is adopted, encompassing supportive ventilation, physical protection and vigilant monitoring. Antiepileptic medications such as lorazepam, diazepam, phenobarbital, phenytoin (PHT), and propofol should be administered judiciously based on the type, severity and duration of seizures.^[^ ^159^ ^]^ For drug‐resistant epilepsy, alternative treatments may include surgical interventions and specialized dietary regimens, such as the ketogenic diet.^[^ ^253^ ^]^ Status epilepticus (SE), characterized by continuous and repetitive seizures, is a medical emergency that may lead to severe neurological complications and a short‐term mortality rate ranging from 15% to 20%.

Nanomedicines have been developed to enhance the treatment of epilepsy.^[^ ^253^ ^]^ Clinical antiepileptic drugs such as PHT, carbamazepine (CBZ), and lamotrigine (LTG) have been encapsulated within various nanoplatforms to gain several benefits, including improved bioavailability, enhanced BBB permeation, avoidance of first‐pass liver metabolism, reduced drug resistance, and mitigation of side effects.^[^ ^253^, ^255^, ^256^, ^257^ ^]^ For instance, conventional administration of CBZ is hampered by several factors, including autoinduction of liver enzymes leading to rapid metabolism, a short half‐life time, a narrow therapeutic window, susceptibility to side effects, and efflux transporters limiting brain access. To address these challenges, intranasal delivery of CBZ‐loaded carboxymethyl chitosan (CMC) NPs (denoted ‘CBZ‐NPs’) has been proposed as a solution. CBZ‐NPs were fabricated by titrating CBZ‐contained acetone into CMC aqueous solution followed by sonication and solvent evaporation. PK results indicated that nasal delivery of CBZ‐NPs resulted in faster and more pronounced CBZ distribution in the brain, reaching levels 150% of those in the plasma. Compared to CBZ solutions, the enhanced bioavailability of CBZ‐NPs can be attributed to the mucoadhesive properties of carboxymethyl chitosan NPs that improve its brain distribution.^[^ ^258^ ^]^ Likewise, another chitosan‐based mucoadhesive microemulsion was developed by a water titration‐vortex method to enhance the nose‐to‐brain delivery of diazepam for the initial treatment of emergent epilepticus. The optimized formulation (MME2) contained mucoadhesive chitosan and exhibited a particle size of 96.45 nm and a zeta potential of 13.52 mV. Nasal administration of MME2 significantly enhanced the brain transport of diazepam, resulting in prolonged latency periods of minimal clonic seizures (MCS) and generalized tonic‐clonic seizures (GTCS).^[^ ^257^ ^]^

Besides intranasal administration, the aforementioned BBB‐penetrating strategies have also been employed to enhance the brain delivery of antiepileptic drugs. Lacosamide (LCM), an approved antiepileptic drug for drug‐resistant generalized epilepsy, may be ineffective in atypical absence seizures due to limited brain concentration. Intravenous injection of LCM‐loaded glucose‐coated Au NPs resulted in sustained high‐level accumulation of LCM in the brain parenchyma owing to the interaction between the glucose coating and GLUT1 transporters at the BBB, and the downregulation of P‐gp mediated by the Au NPs. The improved LCM brain concentration decreased amplitude and frequency of electroencephalograph (EEG), alleviated anxiety‐like behavior and reduced the number of epileptic mice under a significantly lower LCM dose.^[^ ^193^ ^]^ Furthermore, Zhao et al. constructed a brain‐targeting peptide (TGN)‐anchored hepatitis B core protein (HBc) nanocages (NCs) to deliver PHT, marked as PHT@TGN‐HBc for treating refractory epilepsy. Protein engineering was adopted for the preparation of self‐assembling TGN‐HBc NCs, which included gene design and synthesis, vector transfection into *E. coli*, protein expression and purification. The resulting TGN‐HBc NCs were disassembled with urea and mixed with β‐CD‐complexed PHT, followed by reassembling into PHT@TGN‐HBc. This nanoformulation improved PHT's solubility, circulation time, brain accumulation, neuron‐targeting ability and sustained drug release. In a mouse model of pilocarpine‐induced acute epilepsy, PHT@TGN‐HBc (0.2 mg PHT kg^−1^) exhibited antiepileptic effects comparable to a 100‐fold higher dose of free PHT (20 mg kg^−1^), greatly mitigating the striking features of epilepsy in terms of latency of general seizures, duration of GTCS, and the number of seizures in 1 h. Remarkably, the low‐dose PHT@TGN‐HBc (0.2 mg PHT kg^−1^) relieved seizures in PHT‐resistant mice, while high doses of free PHT proved ineffective.^[^ ^259^ ^]^ More recently, to overcome PHT resistance, PHT was incorporated into calcium phosphate NPs modified with PEGylated BBB‐penetrating TAT peptide, termed CaP@PHT‐PEG‐TAT. CaP@PHT was fabricated by biomineralization, which was further incubated with PEG‐TAT for surface modification. Intravenous injection of CaP@PHT‐PEG‐TAT effectively delivered PHT to epileptic neurons, resulting in the attenuation of acute seizures, neuronal injury, astrogliosis and microgliosis. The reversal of PHT resistance is achieved through Ca^2+^ release following lysosomal degradation of CaP@PHT‐PEG‐TAT, which downregulated P‐gp expression and enhanced the brain retention of PHT.^[^ ^190^ ^]^

**Figure 9:**
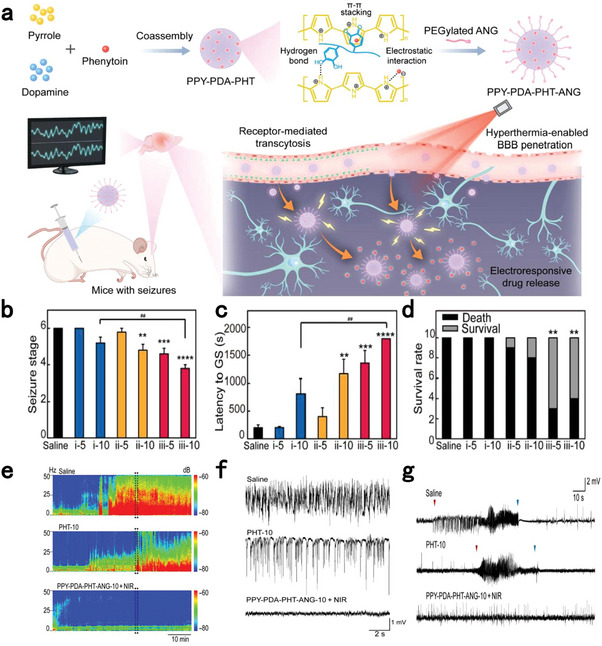
Scheme a) of synthesizing electroresponsive brain‐targeting PPY‐PDA‐PHT‐ANG and its protective effects against epilepsy with NIR irradiation; b) Seizure stage of epileptic mice, c) latency to GS, and d) survival rate in the PTZ‐induced seizure model after different treatments. N = 10; e) Corresponding power spectral analysis of different groups during pilocarpine‐induced SE; Representative EEGs recorded from the hippocampus of rats after different treatments in the pilocarpine‐induced SE model f,g) KA‐induced chronic epilepsy model. Red and blue triangles indicate the start and the end of one complete GS. Reproduced with permission.^[^ ^178^ ^]^ Copyright 2016, Wu et al., open access.

Although brain‐targeting nanomedicines can enhance the brain delivery of antiepileptic drugs, most of these nanoplatforms lack the capability for on‐demand drug release. Since seizures often occur in an abrupt, unpredicted manner and may last for a relatively long time, ideal nanocarriers should possess the ability to promptly control seizure spreading and maintain sustained drug release to suppress continuous seizures.^[^ ^189^ ^]^ In this context, Wu et al. developed a NIR‐ and electro‐responsive nanoplatform with synergistic BBB‐penetrating and on‐demand drug‐releasing ability for treating seizures (**Figure** **9**). In the presence of polyvinyl alcohol (PVA, a stabilizing agent) and ammonium persulfate (oxidant), dopamine, pyrrole copolymerized into nanoparticles, which encapsulated PHT through hydrogen bonds, hydrophobic interactions, and π interaction. Owing to the distinct NIR‐II absorbing ability of PDA, PHT‐PPY‐PDA can effectively convert NIR‐II light into heat to facilitate BBB penetration. The surface of PHT‐PPY‐PDA was further chemically conjugated with thiol‐PEGylated brain‐targeting peptide ANG, which enhances BBB penetration through interacting with the LDLR1. Moreover, PHT‐PPY‐PDA exhibited a 125‐fold escalation in conductivity compared to pure PPY, enabling rapid release of PHT upon the onset of epileptic discharges. In an acute seizure model induced by pentylenetetrazole, treatment with PHT‐PPY‐PDA‐ANG (10 mg PHT kg^−1^) and NIR irradiation significantly reduced seizure severity, increased latency to GS and improved survival rates. In a pilocarpine‐induced epilepsy model mimicking clinical SE, sustained release of PHT‐PPY‐PDA‐ANG‐10 + NIR remarkably mitigated SE severity with a low incidence of GS and no SE behavior. Conversely, mice treated with 10 mg kg^−1^ PHT showed no therapeutic effects on SE. Furthermore, the kainic acid (KA)‐induced chronic epilepsy model, representing a recurrent and spontaneous seizure model, was also used to assess its therapeutic potential. The results indicated that PHT‐PPY‐PDA‐ANG‐10 + NIR reduced spontaneous seizures regarding GS number, duration and severity, whereas PHT‐10 alone showed no effects. However, all groups showed limited therapeutic effects on nonconvulsive focal seizures (FS), as PHT is primarily effective against GS rather than FS.^[^ ^189^ ^]^

In addition to nanocarriers loaded with clinical antiepileptic drugs, various nanodrugs with antioxidative and anti‐inflammatory bioactivity have proven their effectiveness in seizure treatment. These include herbal molecules (e.g., berberine, resveratrol, and rutin‐loaded NPs),^[^ ^195^, ^260^, ^261^ ^]^ antioxidative nanozymes (e.g., selenium NPs),^[^ ^262^ ^]^ and extracellular vesicles containing antioxidative microRNA.^[^ ^263^ ^]^ These nanodrugs primarily function by inhibiting and restoring neurological damages and functional impairments,^[^ ^263^ ^]^ suppressing oxidative stress, inflammation, hyperexcitability and apoptosis.^[^ ^195^, ^260^ ^]^ Integrating clinical antiepileptic drugs with antioxidative and anti‐inflammatory molecules in nanoplatforms with BBB‐penetrating and on‐demand drug‐releasing capabilities may shed light on the effective treatment of epilepsy.

### 5.2 Anesthesia‐Related Traumatic Nerve Injury

Despite the infrequency of spinal cord injury (SCI) during subarachnoid and epidural blockades, it is of significant importance to develop effective treatments for SCI given its devastating consequences.^[^ ^88^ ^]^ The pathological progression of SCI tends to follow three stages: 1) primary physical trauma resulting in membrane damage, death of neurons and glial cells and blood vessel rupture; 2) oxidative stress and activation of inflammatory cells to clear cell debris; 3) formation of glial scars that prohibit further infection and neuron regrowth.^[^ ^17^, ^264^, ^265^ ^]^ Clinically, neuroprotective and anti‐inflammatory medications have been employed to alleviate cell death and inflammation for SCI. However, these drugs demonstrate questionable therapeutic efficacy and potential severe toxicity as their accumulation in the spinal cord is minimal, even when administered at high dosages due to the brain‐spinal cord barrier.^[^ ^264^ ^]^ As for treating late‐stage SCI, the transplantation of stem cells and supportive glial cells for tissue regeneration shows promise. However, their clinical trial outcomes were disappointing, possibly due to the survival rates, differentiation and bioactivity maintenance of the transplanted cells.^[^ ^17^, ^266^, ^267^ ^]^ Consequently, nanotechnology‐integrated approaches have been employed to develop multifunctional nanomedicines or scaffolds for treating SCI and promoting spinal cord regeneration.^[^ ^17^, ^19^, ^268^ ^]^

By virtue of the bioactivities of nanoplatforms and/or their loaded drugs, nanomedicine with proper surface modification can target the damaged spinal cord and exert membrane‐protective, antioxidative, anti‐inflammatory and neuroprotective effects for SCI management. For instance, Shi et al. prepared self‐assembling PEG–poly (D, L‐lactic acid) (mPEG–PDLLA) micelles using a membrane dialysis method. mPEG–PDLLA with membrane‐sealing capability has been shown to effectively ameliorate compression‐induced axonal membrane damage, restore compound action potential of ex vivo spinal tissue, recover locomotor function and reduce the lesion volume and inflammation severity in SCI rats.^[^ ^265^ ^]^ Additionally, anti‐inflammatory and antioxidative nanomedicines such as selenium NPs, cerium oxide NPs, and bioactive molecules (e.g., resveratrol, tanshinone IIA, astragalus polysaccharides, curcumin, estrogen and minocycline)‐loaded NPs were revealed to mitigate SCI and promote spinal cord functional recovery by inhibiting oxidative stress, inflammatory response, cell death, or gliosis.^[^ ^269^, ^270^, ^271^, ^272^, ^273^ ^]^ Furthermore, versatile nanodrugs have exhibited direct effects on neuroprotection, NSC proliferation and differentiation, neuronal cell outgrowth, axonal alignment, neural regeneration, etc.^[^ ^233^, ^274^, ^275^, ^276^ ^]^ This neuroprotective property of nanodrugs is derived from encapsulated compounds (e.g., neurotrophins, nerve growth factor, brain‐derived neurotrophic factor, RA, minocycline or adenosine),^[^ ^273^, ^277^, ^278^ ^]^ and/or from inherent properties (e.g., graphene family, fullerene, exosomes).^[^ ^233^, ^275^, ^279^ ^]^

**Figure 10:**
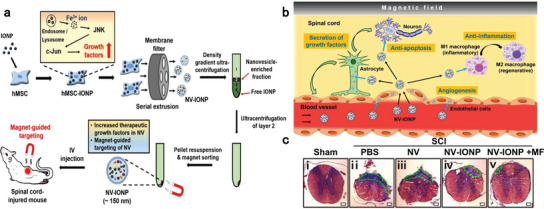
Schematic illustration of a) preparing IONP‐incorporated exosome mimetics nanovesicle (NV‐IONP) and magnet‐guided in vivo targeting to the injured spinal cord, and b) therapeutic effects of NV‐IONP on various cells (i.e., endothelial cells, astrocytes, neurons, and macrophages) in injured spinal cord; c) Masson's trichrome‐stained transverse sections of lesion core tissues. The dotted area indicates the fibrotic tissue area. Bars = 100 µm. Reproduced with permission.^[^ ^273^ ^]^ Copyright 2018, ACS publication.

The therapeutic potential of nanodrugs for SCI can be further enhanced by improving their targeting capability to the damaged spinal cord and the penetration efficacy of blood‐spinal cord barrier. Similar to overcoming the BBB, cell‐penetrating peptides (e.g., TAT), targeting ligands and biomimetic membranes have been integrated to target inflammatory spinal cord sites.^[^ ^273^, ^280^, ^281^, ^282^, ^283^ ^]^ For example, adhesion molecules (e.g., E‐selectin) and nutrient transporters/receptors (e.g., nAChR) are highly expressed in the endothelial cells of the blood‐spinal cord barrier, which were reported to interact with sialic acid/macrophage membrane proteins and nutrient analogs (e.g., L‐arginine), respectively, for enhanced targeting efficiency.^[^ ^279^, ^281^, ^284^ ^]^ Furthermore, physical stimuli such as ultrasound and magnetic field can promote nanodrugs’ spinal cord targeting ability. For example, human mesenchymal stem cells (hMSCs) were treated with IONP to yield IONP‐incorporated exosome‐mimetic nanovesicles (NV‐IONP). Specifically, NV‐IONP were prepared by serial extrusion of hMSC‐IONP, followed by density gradient ultracentrifugation to collect NV‐enriched fraction, and finally magnetic sorting to isolate NV‐IONP. NV‐IONP not only exhibited remarkable accumulation in the damaged spinal cord under magnetic guidance but also contained various growth factors regarding angiogenesis, anti‐apoptosis, neurotrophic and anti‐inflammation generated by IONP‐treated hMSCs. Consequently, intravenous injection of NV‐IONP attenuated neurodegeneration, astrogliosis and neuroinflammation, preserved the spinal cord structure with a reduced fibrotic area and promoted functional recovery following SCI (**Figure** **10**).^[^ ^282^ ^]^

Owing to their versatile electronic, mechanical, physicochemical and engineering properties, nanomaterials are desirable building blocks for neural tissue engineering scaffolds. Ideal scaffolds provide suitable mimetic biological, physical and chemical cues for endogenous or exogenous stem cells to guide their migration, adhesion, proliferation and differentiation, thus facilitating neural tissue regeneration.^[^ ^17^, ^19^, ^274^ ^]^ In this respect, nanomaterials have been integrated into scaffolds in various forms such as nanofilms, nanomembranes, nanofibers, nanosponges, and hydrogels. These scaffolds typically offer multiple features for SCI repair, including surface roughness and functionalization apt for cell adhesion, porosity for substance exchange, chemical gradients and spatial patterns for aligned neural activity, flexibility and viscoelasticity for cell support and neurite extension, electroconductivity for bioelectrical signal propagation, and encapsulated cells/exosomes/growth factors/drugs for alleviating damage and improving tissue regeneration.^[^ ^17^, ^19^, ^285^, ^286^ ^]^ Various natural polymers (e.g., collagen, elastin, gelatin, hyaluronic acid, chitosan and silk), synthetic polymers (e.g., PEG, PLGA, poly(L‐lactic acid)(PLLA)), self‐assembling peptides and their hybrids have been employed in fabricating neural scaffolds. In‐depth discussion regarding the techniques and materials used to develop neural scaffolds can be found in these publications.^[^ ^274^, ^286^, ^287^ ^]^

**Figure 11:**
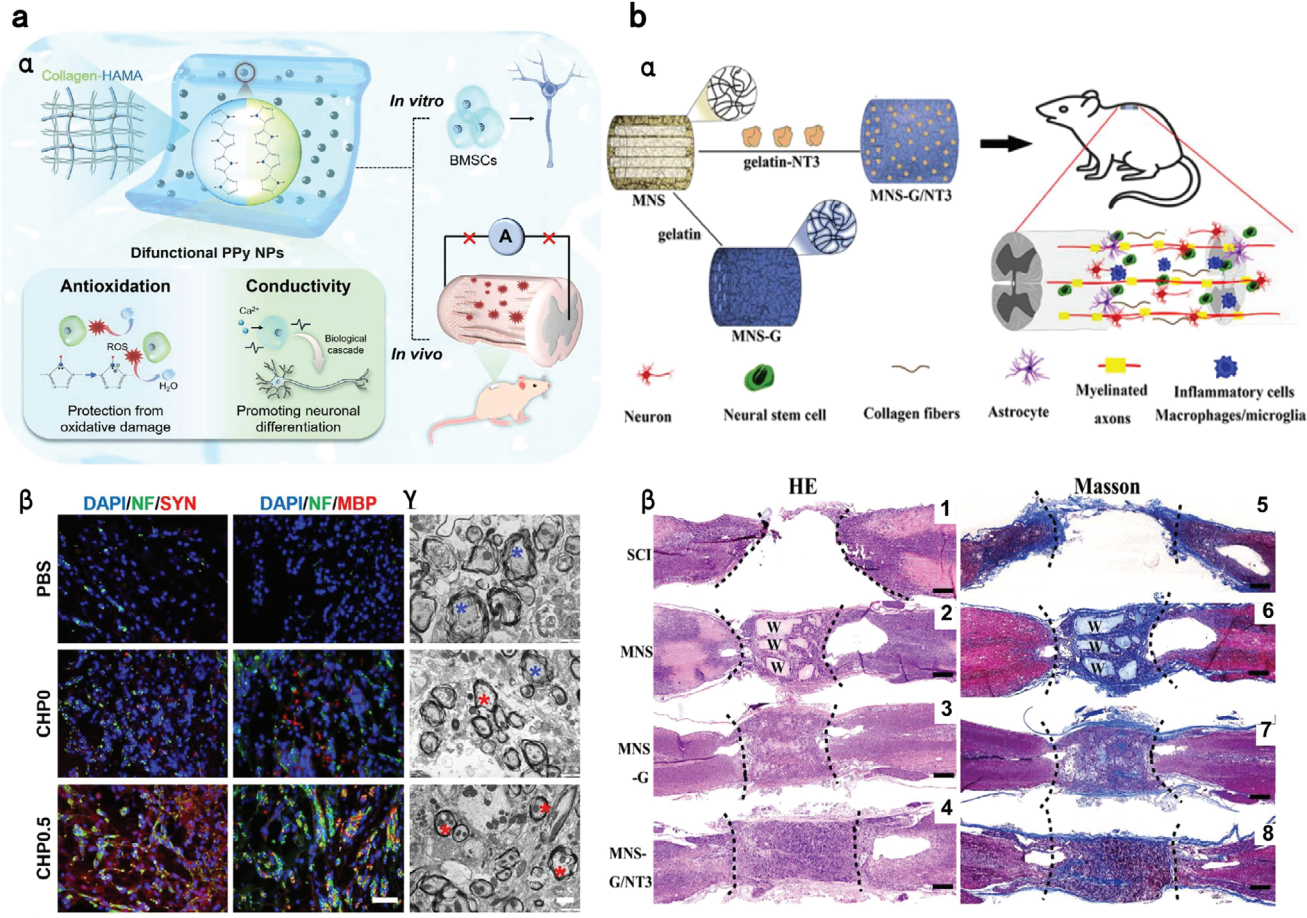
a) Scheme of (α) difunctional PPy NP‐embedded collagen−HAMA hybrid hydrogel for in vitro BMSC neuronal differentiation and in vivo SCI repair; (β) Costaining NF (green)/SYN (red)/DAPI (blue) and NF (green)/myelin basic protein (MBP) (red)/DAPI revealed the formation of synapse and myelinated nerve fibers, respectively, under different treatment, scale bar: 50 µm; (γ) TEM images of the ultrastructure of myelin sheaths in the cross sections of the lesion sites, in which blue asterisks denote loose myelin sheaths and red asterisks denote compact myelin sheaths, scale bar: 1 µm. Reproduced with permission.^[^ ^280^ ^]^ Copyright 2021, ACS publication. b) Scheme of (α) the structure and therapeutic mechanism of MNS‐G/NT3 for SCI treatment; Cavity and collagen scar formation at the lesion sites, characterized by H&E (β_1‐4_) and Masson trichrome staining(β_5‐7_), respectively, 8 weeks post‐implantation. “W” denotes the side wall fragments of the nanofibrous scaffolds in (β_2_) and (β_6_), scale bar: 500 µm. Reproduced with permission.^[^ ^18^ ^]^ Copyright 2020, ACS publication.

Notably, electroconductive nanomaterial‐integrated scaffolds have been revealed to enhance SCI repair and recovery of sensory and motor functions, particularly in conjunction with external electrical stimulation.^[^ ^288^, ^289^, ^290^ ^]^ These electroconductive materials encompass carbon‐based nanomaterials (e.g., graphene, carbon nanotube), 2D nanosheets (e.g., MoS_2_, MXenes), organic polymers (e.g., polypyrrole, polyaniline) and inorganic nanomaterials (e.g., Au NPs) and their derivatives and composites.^[^ ^289^, ^290^, ^291^, ^292^, ^293^ ^]^ For instance, Yu et al. developed an injectable, electroconductive, adhesive and self‐healing hydrogel composed of polyvinylpyrrolidone (PVP)/phytic acid (PA)/Mxenes (denoted as ‘PPM’) for SCI restoration. PPM was fabricated by uniformly mixing PVP, PA and Mxenes followed by pH adjustment to 7.4 and continuous stirring. PPM implantation into the lesions alleviated SCI regarding functional and structural recoveries, which were mediated by PPM‐enhanced angiogenesis, axonal regeneration, remyelination and activation of Ca^2+^ channels.^[^ ^293^ ^]^ Other hydrogels with appropriate mechanical, electrical and biological properties for SCI repair include hydrogel made of anti‐inflammatory diacerein‐grafted multi‐arm PEG and GO nanosheets,^[^ ^294^ ^]^ molybdenum sulfide/graphene oxide/polyvinyl alcohol nanocomposite (MoS_2_/GO/PVA) hydrogel with ROS‐scavenging and inflammation‐mitigating effects,^[^ ^291^ ^]^ and polypyrrole NPs embedded in collagen/hyaluronan hybrid hydrogel (collagen‐HAMA‐PPy),^[^ ^289^ ^]^ etc. For example, Wu et al. first fabricated antioxidant and electroconductive PPy NPs based on the complex between PVA and ferric ions and the UV cross‐linkable HAMA polymer by the esterification of HA. The preformed PPy NPs and HAMA were uniformly mixed with collagen and BMSCs, and then transferred into sterile cylindrical molds, followed by 37 °C incubation and UV irradiation to form BMSCs‐laden collagen‐HAMA‐PPy hydrogel. Remarkably, under external electrical stimulation, the BMSCs‐laden hydrogel can not only provide a neuroprotective niche by eliminating ROS but also promote neuronal differentiation by facilitating intercellular bioelectric transfer (**Figure** **11**a).^[^ ^289^ ^]^

In addition to multifunctional hydrogel scaffolds, nanofiber and nanofiber‐hydrogel scaffolds have garnered substantial interest for neural tissue engineering due to their superior capability to mimic extracellular matrices.^[^ ^18^, ^295^ ^]^ Sun et al. fabricated neurotrophin‐3‐loaded gelatin‐modified PLLA multichannel nanofibrous scaffolds (MNS‐G/NT3) for SCI treatment. MNS‐G/NTS preparation was conducted in a mold through thermally induced phase separation of PLLA, followed by the modification of gelatin cross‐linked by genipin in the presence of NT‐3. The neuronal differentiation of in vitro seeded NSCs and synaptic formation were enhanced synergistically by gelatin modification and NT‐3 integration. In a completely transected SCI model, implantation of MNS‐G/NT3 in the defected sites significantly reduced cavity formation, collagen and glial scar deposition, and inflammatory infiltration. It also improved the recruitment and neuronal differentiation of endogenous NSCs, enhanced the regeneration of functional neurons and axons, axonal outgrowth, nerve fiber regeneration and remyelination, thus efficiently restoring the locomotor function of transected SCI rats (Figure 11b).^[^ ^18^ ^]^ Similarly, nerve growth factor‐loaded aligned silk fibroin nanofiber hydrogel with proper softness and hierarchical topography facilitated the structural and functional restoration of SCI by guiding cell migration, neural differentiation and angiogenesis.^[^ ^295^ ^]^ However, it is worth noting that the therapeutic potential of these neural scaffolds should be evaluated in regional anesthesia‐induced SCI models.

As aforementioned, peripheral nerve blocks pose a low risk of causing physical nerve damage, primarily resulting from practice misconduct. In contrast to the limited regenerative capability of the spinal cord, peripheral nerves with moderate self‐restoring ability can instinctively regenerate following minor nerve injuries.^[^ ^17^ ^]^ In clinical practice, gluing or suturing without nerve grafting is effective for treating nerve transections < 5 mm, whereas nerve grafting and/or nerve guidance conduits are adopted for managing long‐gap (>10 mm) nerve injuries.^[^ ^17^, ^274^ ^]^ Therefore, in the context of regional/local anesthesia‐induced toxicity, long‐gap peripheral nerve injuries seem unlikely to occur and are beyond the scope of this manuscript. Briefly, nanotechnology‐facilitated therapeutics and tissue engineering scaffolds with or without external stimulus can provide topological, mechanical, electroconductive, and biochemical cues to bridge peripheral nerve lesions, promote axon regeneration, elicit Schwann cell repair‐supportive functions, and alleviate neuroinhibitory environments characterized by oxidative stress and inflammation.^[^ ^19^, ^296^, ^297^ ^]^

### 5.3 Anesthetics‐Related Acute Hepatitis

Surgery with anesthesia often leads to hepatic dysfunction, ranging from a transient and mild increase in transaminase levels to hepatitis, cirrhosis, fulminant hepatic failure and even death. Remarkably, halothane anesthesia is associated with an ≈20% incidence of fulminant hepatitis with poor prognosis and high mortality. Although modern halogenated anesthetics such as enflurane, desflurane and sevoflurane induce hepatotoxicity less frequently and severely, cases of acute hepatitis associated with these modern halogenated anesthetics can still be fatal and warrant significant attention.^[^ ^37^ ^]^ In essence, halogenated anesthetics‐related hepatitis is an autoimmune liver disorder triggered by neoantigens, which are generated by the covalent binding of TFA (a metabolite of halogenated anesthetics) to hepatic proteins.^[^ ^37^, ^298^ ^]^ Neoantigens can be primed by Kupffer cells to activate innate and adaptive immune responses. Various immune cells including Kupffer cells, neutrophils, natural killer T cells, eosinophils, and helper, effector and regulatory T cells were reported to crosstalk via cytokines, chemokines, and sex hormones, affecting the induction and progression of fulminant hepatitis.^[^ ^298^, ^299^, ^300^, ^301^, ^302^ ^]^ Clinical manifestations of halogenated anesthetics‐induced hepatitis closely resemble those of acute hepatitis caused by viral infections, hypoxia and various hepatotoxic drugs, making its diagnosis an exclusive process.^[^ ^37^ ^]^ Current clinical treatments for autoimmune hepatitis primarily rely on supportive care and immunosuppressive therapy such as corticosteroids, mycophenolate mofetil, and cyclophosphamide.^[^ ^302^ ^]^ However, these treatments often have severe side effects due to their narrow therapeutic windows and lack of liver‐targeting ability.

**Figure 12:**
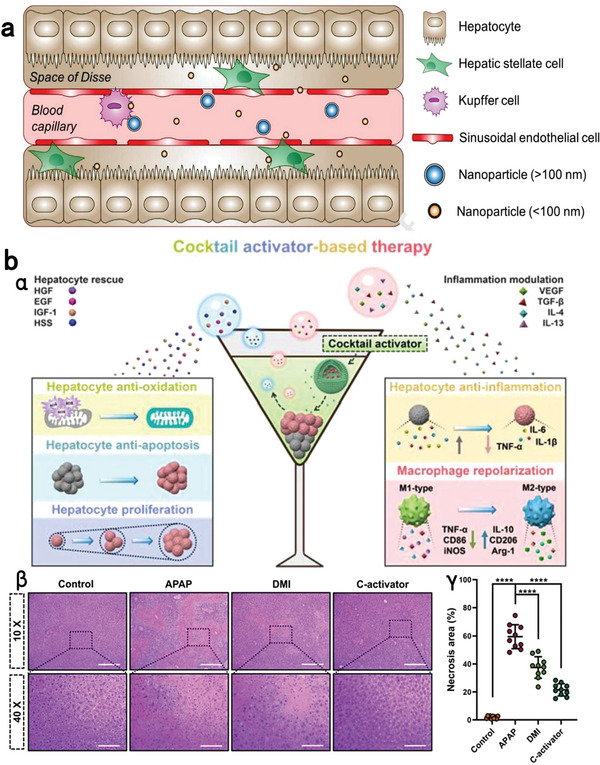
Nanomedicines facilitating liver‐ and kidney‐targeted accumulation are promising candidates for treating acute hepatotoxicity and nephrotoxicity caused by anesthetics. a) The structure of liver sinusoid; nanoparticles of different sizes can target Kupffer cells and extravasate into the Disse space to target hepatocytes. Reproduced with permission.^[^ ^15^ ^]^ Copyright 2020, Elsevier publication. b) Scheme of (α) the cocktail activator‐based therapy realizing hepatocyte rescue and inflammation modulation. (β) Representative images of H&E‐stained livers in different groups. Scale bars: 400 µm, top; 100 µm, bottom. (γ) Quantification of liver necrosis areas by ImageJ analysis (n = 10; mean ± SD). ^*^ *p* < 0.05; ^**^ *p* < 0.01, ^***^ *p* < 0.0001 compared with the APAP group by one‐way ANOVA test. Reproduced with permission.^[^ ^303^ ^]^ Copyright 2022, Shenyang Pharmaceutical University, open access.

Owing to the liver's abundant blood supply, unique physiological structure (e.g., the Disse space) and cellular components (e.g., Kupffer cells), nanocarriers with specific physicochemical features can rapidly deliver large quantities of therapeutics to the liver and accumulate there for an extended duration through passive and/or active targeting mechanisms. In general, liver‐targeting nanoplatforms often have small apparent sizes (<100 nm), which are smaller than the width of liver sinusoidal fenestrations (100–200 nm). This feature allows them to enter the Disse space and interact with hepatocytes. Larger NPs (>100 nm) can be internalized by Kupffer cells or sinusoidal endothelial cells (**Figure** **12**a).^[^ ^15^ ^]^ Besides size‐based passive targeting, integrating cell‐specific targeting ligands onto the surface of nanomedicine facilitates their active targeting to the liver and even a specific hepatic cell population.^[^ ^15^ ^]^ Refined PK profiles of nanomedicines are particularly desirable for treating halogenated anesthetics‐induced fulminant hepatitis.

To the best of our knowledge, no research has yet explored the therapeutic potential of nanomedicines for halothane hepatitis. Nevertheless, according to the pathological mechanisms underlying halothane hepatitis and the therapeutic efficacy of various nanodrugs for addressing other forms of acute hepatitis, the modulation of Kupffer cells and hepatocytes holds significant promise for alleviating halothane hepatitis. For instance, a polythiolated and mannosylated human serum albumin (SH‐Man‐HSA) was fabricated by the reaction between 2‐iminothiolane and recombinant Man‐HAS generated by a yeast system. The SH‐Man‐HSA with the ability to selectively and rapidly deliver thiol to CD68+ Kupffer cells through interactions with mannose receptors, rescued concanavalin‐A and acetaminophen‐induced acute hepatitis by inhibiting ROS generation.^[^ ^303^ ^]^ A panel of selectin‐binding glycopolymers exhibited delicate interactions with Kupffer cells, which further intensely impact leukocyte infiltration, macrophage activation, and the progression of hepatitis.^[^ ^304^ ^]^ Palmitic acid‐modified serum albumin (PSA) have been reported to efficiently bind to scavenger receptors on the surface of activated Kupffer cells to deliver NF‐κB inhibitor (i.e., Schisandrin B, SchB). PSA was synthesized by the reaction between the lysine residue of HAS and PA‐NHSE. The resulting PSA was further mixed with SchB under sonification followed by evaporation to prepare SchB‐PSA NPs. This nanoplatform exhibited rapid liver accumulation and potent immunosuppressive effects, significantly inhibiting hepatic necrosis and improving survival rates.^[^ ^305^ ^]^ Moreover, intravenous injection of siRNA‐encapsulated lipid or polymeric NPs revealed varying degrees of remission in acute hepatitis models by downregulating the expression of various inflammatory factors, including IL‐1β,^[^ ^306^ ^]^ TNF‐α,^[^ ^307^ ^]^ and NF‐κB.^[^ ^308^ ^]^

In addition to inhibiting Kupffer cell‐initiated oxidative stress and immune responses, protecting against hepatocyte death has shown promise. For instance, antioxidative selenium compound L‐Se‐methylselenocysteine (SeMC) was encapsulated into self‐assembling micelles by a film hydration method to form SeMC NPs. The amphiphilic polymers were modified with the hepatocyte‐targeting ligand glycyrrhetinic acid (GA) to yield GA‐SeMC NPs, which displayed enhanced hepatocyte uptake and liver accumulation. The nanodrug was suggested to ameliorate hepatic vacuolization and biochemical abnormality via inhibiting oxidative stress.^[^ ^309^ ^]^ Furthermore, a Ca^2+^ chelator (BAPTA‐AM)‐encapsulated liposome (BAL) was prepared by a thin film hydration‐extrusion method. The BAL was reported to rapidly distribute to the liver and remarkably restore liver health parameters and microcirculation, leading to an ≈70% increase in the survival rate of mice with acute liver failure (ALF). The underlying hepatoprotective mechanisms primarily stem from BAL's ability to clear excess Ca^2+^, resulting in synergistic suppression of hepatocyte necrosis/apoptosis, oxidative stress, and NF‐κB activation.^[^ ^310^ ^]^ Furthermore, a combination therapy that simultaneously mitigates inflammatory and oxidative damage, promotes hepatocyte regeneration and restores the liver microenvironment has been validated as a promising therapeutic strategy for ALF treatment. In this regard, Yin et al. synthesized dimethyl itaconate (DMI)‐loaded liposomes modified with dodecyl gallate (DG), termed as C‐activator, using a reverse‐phase evaporation method. Briefly, DMI‐contained aqueous phase was added dropwise into DG‐contained oil phase, followed by sonication, evaporation and hydration. In the C‐activator, DMI served as an antioxidant to activate NRF2 and DG induced the expression of various growth and repair factors to promote hepatocyte expansion and macrophage repolarization into the M2 repair phenotype (Figure 12b).^[^ ^311^ ^]^

### 5.4 Anesthetics‐Related Acute Kidney Injuries

In general, the majority of anesthetics have no obvious negative effects on renal function. Several anesthetics, especially propofol and amide local anesthetics, have been suggested to protect against perioperative renal ischemia/reperfusion (IR) injury.^[^ ^312^ ^]^ However, the impact of modern halogenated anesthetics, particularly sevoflurane, on renal function remains a topic of controversy, as both their potential protective effects against renal IR injury and nephrotoxicity resulting from metabolites have been reported.^[^ ^42^, ^312^, ^313^ ^]^ Unlike their prototype methoxyflurane, clinical dosages of isoflurane, enflurane and halothane are associated with minimal increases in blood fluoride ion levels, thus presenting negligible nephrotoxicity. However, sevoflurane leads to a relatively high concentration of blood fluoride ions and reacts with the absorbent material to generate nephrotoxic compound A, thus carrying a higher risk of acute renal damage.^[^ ^42^, ^312^ ^]^

Nanomedicines offer substantial opportunities for addressing anesthetics‐associated acute nephrotoxicity due to favorable properties, including a large surface area, high drug loading capacity, ease of engineering, versatile bioactivities, kidney‐targeting capabilities and sustained release profiles.^[^ ^16^, ^314^ ^]^ The histological structure and abundant blood supply of the kidney endow it with inherent retention toward specific nanodrugs, which are typically characterized by relatively small sizes and balanced surface charges. Theoretically, ultrasmall nanodrugs with sizes of 3–7 nm could traverse the glomerular filtration apparatus to reach the apical side of tubular epithelial cells. The glomerular filtration process involves three barriers, including glomerular endothelial cells with a normal fenestration width of 60–80 nm, glomerular basement membrane (i.e., a dense extracellular matrix with small pore sizes of 3 nm) and podocytes with normal filtration slits of 32 nm.^[^ ^16^, ^314^ ^]^ Notably, the glomerular filtration apparatus becomes more permeable in the injured kidney, allowing larger NPs (40 nm or even 110 nm) to reach renal tubules.^[^ ^16^, ^314^, ^315^, ^316^ ^]^ Further surface modification of nanodrugs with high‐affinity ligands facilitates active targeting to specific renal cell types.^[^ ^16^, ^314^ ^]^ In the context of acute nephrotoxicity, including sevoflurane‐induced renal toxicity, targeting tubular epithelial cells and glomerular endothelial cells is highly desirable as sudden tubular injury and endothelial dysfunction are the primary pathological mechanisms.^[^ ^314^ ^]^ Detailed discussion of specific renal cell‐targeting strategies and nanotherapeutics for treating acute kidney injuries can be found in these reviews.^[^ ^16^, ^314^, ^317^ ^]^

**Figure 13:**
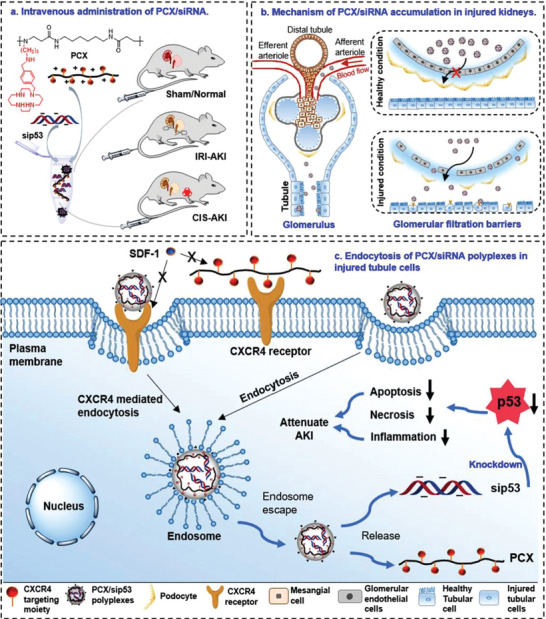
Scheme of selective accumulation and proposed mechanisms of PCX/sip53 polyplexes in the treatment of acute kidney injuries. Reproduced with permission.^[^ ^308^ ^]^ Copyright 2022, Elsevier publication.

To our knowledge, only one study has directly investigated the efficacy of nanodrugs in treating sevoflurane‐induced renal toxicity. This study revealed that pretreatment with fullerenol mitigated sevoflurane‐induced nephrotoxicity by inhibiting oxidative stress.^[^ ^42^ ^]^ Similarly, selenium‐doped carbon quantum dots (SeCQDs) synthesized by a hydrothermal method exhibited good kidney accumulating and potent ROS scavenging ability, resulting in remarkable protection against rhabdomyolysis‐ and cisplatin‐induced acute nephrotoxicity.^[^ ^315^ ^]^ In addition to mitigating oxidative stress, sequestering fluoride ions and reducing fluoride ions‐related toxicity may hold promises for treating acute kidney injuries. In this regard, PLGA nanocapsules was prepared via a emulsion–diffusion–evaporation method to co‐delivery the antioxidant (catechin hydrate) and fluoride chelator (sodium metaborate). The nanostructure significantly reduced the fluoride levels in the liver, brain and kidney by over 50%, thereby alleviating fluoride‐induced hepatic, brain and renal toxicity. This therapeutic efficacy can be attributed to the enhanced antioxidant defense system and continuous elimination of fluoride ions through the sustained release of chelators.^[^ ^318^ ^]^ Moreover, kidney‐targeted nanodrugs with anti‐inflammatory and anti‐apoptosis/necrosis properties have demonstrated commendable efficacy in treating other nephrotoxicity and may serve as promising candidates for addressing sevoflurane‐induced kidney injury. For example, Tang et al. developed renal tubule‐targeting polyplexes (PCX/sip53) composed of polymeric chemokine receptor CXCR 4 antagonists (PCX) and siRNA against the p53 protein. The linear PCX was first synthesized by Michael addition copolymerization of phenylene‐cyclam derivative with hexamethyle‐nebisacrylamide, which formed electrostatic complex with negatively charged sip53. As injured tubule cells often overexpress CXCR 4 to recruit and activate inflammatory leukocytes, PCX integration endows PCX/sip53 with high accumulation in the renal tubule and anti‐inflammatory effects. PCX/sip53 can also effectively downregulate the overexpressed p53 protein to inhibit tubular cell apoptosis and necrosis. Consequently, intravenous injection of PCX/sip53 remarkably ameliorated tubule injury with reduced severity in tubule dilation, loss of brush border and cell apoptosis/necrosis (**Figure** **13**).^[^ ^316^ ^]^

### 5.5 MH

Dantrolene sodium is the frontline therapy for MH treatment, which is a fulminant and fatal complication triggered by frequently used volatile anesthetics and Sch. However, the reconstitution and intravenous administration of traditional dantrolene sodium solution is a time‐consuming process due to its poor water solubility. This characteristic can potentially compromise the effectiveness of dantrolene sodium treatment for MH, as any delay in MH management increases the risks of morbidity and mortality.^[^ ^5^, ^319^ ^]^ Furthermore, the resulting alkaline solution of dantrolene (containing 20 mg of dantrolene and 3 g of mannitol per vial dissolved in 60 mL of water) with a pH 9.5 is highly irritating to peripheral veins and can lead to tissue necrosis if accidentally injected into extravascular regions.^[^ ^5^ ^]^ To address these issues, Eagle Pharmaceuticals developed a novel dantrolene nanosuspension, known as Ryanodex, which received orphan drug approval from the FDA in 2014. Ryanodex, in the form of sterile lyophilized powder, contains dantrolene sodium (250 mg), mannitol (125 mg), povidone (4 mg), polysorbate 80 (25 mg) and pH adjusters per vial.^[^ ^320^ ^]^ Each vial is dissolved in 5 mL of sterile water to yield a suspension with 1.65 mg dantrolene in solution and 48.35 mg within suspending NPs, which disintegrate rapidly in circulation to achieve concentrated and fast delivery of dantrolene.^[^ ^46^ ^]^ The time required to prepare and administer Ryanodex for 80 kg MH‐suspected patients (2.5 mg kg^−1^ or 200 mg dantrolene) is <1 min, which is significantly faster than that of dantrolene solution (≈31.5 min if prepared by one practitioner).^[^ ^319^ ^]^ Prompt treatment of aggressively progressed MH with Ryanodex offers great opportunities to improve therapeutic efficacy.

## 6 Conclusion and Perspectives

Current clinical management of anesthesia‐related toxicity predominantly relies on supportive approaches with mediocre efficacy. Nanomedicine, due to its unique physicochemical and biological characteristics, holds promise in detoxifying overdosed anesthetics and treating related complications. To our knowledge, this manuscript is the first review dedicating to exploring novel, efficient and safe nanotherapeutics for anesthesia‐associated toxicity. Following a brief introduction to commonly used anesthetics and related complications, three nanotechnology‐enhanced strategies for mitigating anesthetics toxicity were discussed along with the prospective research direction. Lastly, the challenges facing the clinical translation of these nanotherapeutics and future endeavors were proposed.

Overall, integrating nanotechnology to counteract anesthesia‐related complications is still in the early preclinical research stage. There are only a few nanotherapeutics that can be used for treating anesthetics toxicity in clinical practice. As mentioned earlier, the lipid nano‐emulsion, that is, Intralipid successfully mitigated LAST in several case reports. However, Intralipid has not been officially approved as the treatment for LAST. Another nanotherapeutics is Ryanodex, which is an FDA‐approved rapid‐release nanosuspension of dantrolene sodium for the prevention and treatment of MH. In fact, the preclinical development of nanotherapeutics for anesthetics toxicity mitigation is also slow. This may result from the understudied pathogenesis of anesthetics‐related toxicity and the lack of proper animal models to resemble the anesthetics toxicity in human.

Common hurdles to the clinical translation of nanotherapeutics for diseases treatment include large‐scale manufacture, maintaining quality consistency within and between batches, and certifying biosafety. Since the physicochemical properties of nanodrugs are closely linked with their therapeutic efficacy and safety, synthesis approaches allowing precise adjustment of nanodrugs’ properties are invaluable. Moreover, balancing the synthesis complexity and multifunction of nanotherapeutics should be thoroughly considered. To achieve facile fabrication of multifunctional nanomedicines, nanomaterials with diverse electronic, structural, physical, chemical, and biological properties can be utilized. As for the toxicological studies of nanomaterials, the impact of biotransformation (e.g., aggregation, degradation, and biocorona formation) on the toxicity of nanotherapeutics and their long‐term toxicity should be meticulously addressed. Unique challenges confronting the clinical implementation of nanodrugs for anesthesia‐associated complications include the lack of preclinical disease models and treatment regimens, and the practical difficulties of conducting randomized, controlled clinical trials.

In conclusion, despite being in its early stage, nanotherapeutics bring considerable opportunities to tackle a large array of anesthetics‐related complications. Nevertheless, successful clinical translation of nanotherapeutics requires additional research endeavors and collaboration between anesthetists, chemists, biologists, and scientists in nanoscience and pharmaceutical sciences.

## Conflict of Interest

The authors declare no conflict of interest.
